# Facility capacity and provider knowledge for cholera surveillance and diarrhoea case management in cholera hotspots in the Democratic Republic of Congo – a mixed-methods study

**DOI:** 10.1080/16549716.2024.2317774

**Published:** 2024-03-05

**Authors:** Mattias Schedwin, Aurélie Bisumba Furaha, Kelly Elimian, Carina King, Espoir Bwenge Malembaka, Marc K Yambayamba, Thorkild Tylleskär, Tobias Alfvén, Simone E Carter, Placide Welo Okitayemba, Mala Ali Mapatano, Helena Hildenwall

**Affiliations:** a Department of Global Public Health, Karolinska Institutet, Stockholm, Sweden; b Astrid Lindgren Children’s Hospital, Karolinska University Hospital, Stockholm, Sweden; c Paediatric Department, Hôpital Provincial Général de Référence de Bukavu, Bukavu, Democratic Republic of the Congo; d Exhale Health Foundation, Abuja, Nigeria; e Department of Epidemiology, Johns Hopkins Bloomberg School of Public Health, Johns Hopkins University, Baltimore, MD, USA; f Center for Tropical Diseases and Global Health, Université Catholique de Bukavu, Bukavu, Democratic Republic of the Congo; g Department of Epidemiology and Biostatistics, Kinshasa School of Public Health, Kinshasa, Democratic Republic of the Congo; h Section Epidemiology, Vetsuisse Faculty, University of Zurich, Zurich, Switzerland; i Centre for International Health, Department of Global Public Health and Primary Care, University of Bergen, Bergen, Norway; j Sach’s Children and Youth Hospital, Stockholm, Sweden; k Public Health Emergencies, UNICEF, Kinshasa, Democratic Republic of Congo; l Programme National d’Elimination du Choléra et de lutte contre les autres Maladies Diarrhéiques, Ministry of Health, Kinshasa, Democratic Republic of Congo; m Department of Nutrition, Kinshasa School of Public Health, Kinshasa, Democratic Republic of Congo; n Department of Clinical Science, Intervention and Technology, Karolinska Institutet, Stockholm, Sweden

**Keywords:** Cholera, conflict, healthcare workers, interventions, Democratic Republic of the Congo

## Abstract

**Background:**

Wider healthcare-strengthening interventions are recommended in cholera hotspots and could benefit other types of diarrhoeal diseases which contribute to greater mortality than cholera.

**Objective:**

Describe facility capacity and provider knowledge for case management of diarrhoea and cholera surveillance in cholera hotspots in the Democratic Republic of Congo (DRC) among health facilities, drug shops, and traditional health practitioners.

**Methods:**

We conducted a sequential exploratory mixed-method study, using focus group discussions, facility audits, and provider knowledge questionnaires during September and October 2022 in North Kivu and Tanganyika provinces, Eastern DRC. Content analysis was used for qualitative data. Quantitative data were summarised by facility level and healthcare provider type. Audit and knowledge scores (range 0–100) were generated. Multivariable linear regression estimated association between scores and explanatory factors. Qualitative and quantitative data were triangulated during interpretation.

**Results:**

Overall, 244 facilities and 308 providers were included. The mean audit score for health facilities was 51/100 (SD: 17). Private facilities had an −11.6 (95% CI, −16.7 to −6.6) lower adjusted mean score compared to public. Mean knowledge score was 59/100 (95% CI, 57 to 60) for health facility personnel, 46/100 (95% CI, 43 to 48) for drug shop vendors and 37/100 (95% CI, 34 to 39) for traditional health practitioners. Providers had particularly low knowledge concerning when to check for low blood sugar, use of nasogastric tubes, and dosing schedules. Knowledge about case definitions for cholera was similar between groups (range 41–58%) except for traditional health practitioners for the definition during an outbreak 15/73 (21%).

**Conclusions:**

Increasing awareness of cholera case definitions in this context could help improve cholera surveillance and control. Increased support and supervision, especially for private providers, could help ensure facilities are equipped to provide safe care. More nuanced aspects of case management should be emphasised in provider training.

## Introduction

In the Democratic Republic of Congo (DRC), an estimated 25 000 children die each year before 5 years of age of diarrhoeal disease, including cholera [1,2]. Cholera is a diarrhoeal disease that can cause major outbreaks, especially affecting children, with high case fatality rates [3]. The DRC was estimated to account for 7% (94 500 to 283 500 cases per year) of global cholera cases and 4–7% (1 900 to 9 500 deaths per year) of cholera deaths in the period 2008–2012, for all age groups [4]. In 2022, the DRC reported 18 517 suspected cholera cases and 294 deaths [5,6] Protect, prevent, and treat measures for cholera are largely the same as for other diarrhoeal diseases. Taking advantage of these synergies in health policies would likely be efficient to lower the overall mortality and morbidity from diarrhoeal diseases. Oral rehydration solution (ORS) and zinc is the cornerstone of diarrhoeal disease treatment in children; however, global coverage remains low, and progress has stagnated [7]. Data from 2017 estimated the coverage of ORS in children with diarrhoea to be 30% and 20% in the cholera endemic DRC provinces of North Kivu and Tanganyika, respectively, [8].

Cholera control activities have historically focused on emergency response, which aim to reduce cases and mortality, rather than prevention of cholera or building capacity and resilience [9]. Current guidelines recommend strengthening the local health system’s capacity for future outbreaks, especially in cholera hotspots [9], and this has also been argued as the best way forward for improving preparedness for outbreaks in the DRC [10].

While the classical vertical system of cholera treatment centres, cholera treatment units, and oral rehydration points are efficient [9], cases are likely to escape detection [4], meaning treatment occurs outside designated facilities. A study performed in the DRC capital Kinshasa in 2018 showed that cholera preparedness in primary healthcare facilities was low [11]. Well-equipped health facilities and well-trained health-care providers are a necessity for cholera surveillance and high-quality case management, and to inform caregivers about correct home treatment and prevention. Pharmacies, drug shops, and traditional health practitioners, are common places where care is sought in the DRC and therefore should also be considered for health policy plans [12]. To develop effective policies and guidelines, understanding the current capacity of the healthcare system to provide care for cholera and other diarrhoeal diseases is important.

Therefore, we aimed to describe the capacity for cholera surveillance and diarrhoeal disease case management in children aged 6–59 months among the three main providers of care in the DRC: public, private and faith-based health facilities; pharmacies and drug shops; and traditional health practitioners. Case management for children 6–59 months was chosen because this group is among the most severely affected by diarrhoeal diseases, including cholera.

## Methods

We conducted a sequential exploratory mixed-methods study (qualitative → quantitative), with focus group discussions (FGDs) followed by facility audits and provider knowledge questionnaires. FGDs, performed in September 2022, captured a broader perspective of shared experiences and reasoning around diarrhoea case management and were used to refine the quantitative data collection tools. Facility audits, performed in October 2022, were used to quantify availability of infrastructure for efficient and safe management of cholera and other diarrhoeal diseases. The knowledge questionnaire focused on domains for safe case management; prevention of diarrhoeal diseases in children; and cholera surveillance measures. We intended to explore the health system that currently exists and therefore included both formal and informal health facilities, pharmacies and drugstores, as well different types of traditional health practitioners. Since formal pharmacies only make up 1% or less of drug dispensing facilities in the DRC [13], we use the term ‘drug shops’ to cover formal pharmacies and drug shops. For ownership, we use the term private meaning ‘private-for-profit’.

### Setting

The study was performed in the provinces of North Kivu and Tanganyika in eastern DRC, which reported 35% (6 451) and 17% (3 189), respectively, of the total suspected cases of cholera reported nationally in 2022 [5,6]. Both provinces are affected by armed conflict. The latest (2018) estimate for under-five mortality is 26 per 1 000 live births for North Kivu and 66 for Tanganyika compared to the national rate of 70/1 000 [8]. Tanganyika declared a new cholera outbreak close to when the quantitative data collection was started; the two health zones in North Kivu reported cases, but under the outbreak threshold level [14]. In general, for the DRC, the state contributes a relatively low percentage of health financing, and both facilities and providers depend on user fees for salaries and procurement of equipment and medication [15]. In view of the humanitarian context, donor subsidies are an important contributor to health financing, in the study setting [16]. These subsidies can, for example, target subsidisation for certain vulnerable patient groups or care and outbreak response for epidemic-prone diseases [17].

### Choice of study areas

One urban and one adjacent rural area classified as cholera hotspots were included from each province. Rural settings close to the provincial capital were chosen for two reasons: (i) they report more cholera cases and are frequently targeted by cholera interventions, and (ii) security issues preclude access to more distant health zones. In North Kivu, the health zone of Karisimbi (in the provincial capital Goma), and the rural health zone of Kirotshe (30 km outside Goma), were selected. In Tanganyika, the study was conducted in the health zones of Kalemie and Nyemba (territory of Kalemie), which together cover the provincial capital Kalemie and extend out to rural areas. More distant parts of the territory of Kalemie and the health zone of Kirotshe were excluded due to insecurity and poor access.

### Focus group discussions

#### Participants

FGDs were performed with medical doctors, nurses, drug shop vendors and traditional health practitioners who all independently treated children with diarrhoea. Discussions were conducted for each cadre separately to minimise dominating-voice effect on participants’ views. A pragmatic decision was made to perform one rural and one urban FGD in each province for each cadre, except for medical doctors where no FGD was performed in the rural setting for Tanganyika province (additional information in Supplementary material 1).

#### Data collection

The FGDs followed a topic guide (Supplementary material 1), which was developed in French by multiple team members, and translated during training into Swahili the lingua franca in the study setting. Piloting was performed through repeated mock FGDs with moderators acting as both participants and moderators. FGDs were performed near the local health office, or in the UNICEF local office, with only moderators and participants present. Discussions took 60–140 minutes, were recorded, transcribed verbatim, and when performed in Swahili, translated into French. Moderators had extensive previous experience in performing FGDs working for the UNICEF Integrated Analytics Cell (CAI) [18].

#### Analysis

All the qualitative data were collected before the analysis started. We used content analysis with an inductive manifest approach, creating condensed meaning units, codes, sub-categories and categories, moving back and forth as needed [19]. Coding was performed manually. Initial analysis was performed by MS. A representative subset of FGDs were read by AF, and the initial analysis was discussed with MS. After some minor adjustments, both authors agreed on the interpretation, and this was then shared with the other authors.

### Quantitative data

#### Participants

For health facilities, sampling took a census approach including all facilities appearing on recently updated facility lists from the provincial health offices. A total of 40 drug shops from each province, conveniently selected and stratified on urban/rural setting, were targeted for inclusion (Supplementary material 1). Convenience sampling was applied for the knowledge questionnaire, selecting one eligible provider in each facility, among all the providers present on the day of the survey. Each provider was assigned a number, and random selection was coded within the data collection tablet. To be eligible the provider needed to provide clinical care independently for children with diarrhoea.

#### Data collection

Data collectors with extensive experience from the UNICEF CAI were trained for two days. Questionnaires were in French, with more technical phrases translated into Swahili during the training session to ensure consistency. Data was collected on tablets using Open Data Kit [20]. The audit questionnaire was performed with the facility manager or head of administration, with input from other relevant staff when needed. The health facility audit questionnaire was adapted from a study in Nigeria [21], which used water sanitation and hygiene (WASH) healthcare facility core indicators [22] and additional questions from the Service Availability and Readiness Assessment tool [23]. The questionnaire was adapted in accordance with the Global Task Force on Cholera Control (GTFCC)’s field manual [24], and the Doctors Without Borders cholera guidelines [25]. Additional questions on prices for ORS and zinc were added.

The knowledge questionnaire was adapted from published examples from Kenya [26], and Nigeria [21], and material based on the World Health Organization’s integrated community case management [27], integrated management of childhood illness [28], emergency triage assessment and treatment guidelines [29], guidelines from the GTFCC [24], and Doctors without borders [25]. The questions were intended to be interpretable for anyone treating children with diarrhoea independently, and knowing the answer is needed to provide high-quality care. The audit and case management questionnaires were piloted in an adjacent urban health zone in North Kivu, with the three provider groups to ensure completeness, clarity, and accuracy.

#### Sample size calculation

An *a priori* sample size calculation was performed to be able to compare the difference in the mean knowledge scores between two provider groups, assuming: a 15-point difference in knowledge score; standard deviation of 23.5 (based on a similar study from Malawi [30]); power of 80% and 5% significance. For this a sample size of 78 respondents was needed.

#### Analysis

Due to inconsistency in health facility-level classifications, we defined all facilities with a medical doctor available as hospitals. Remaining health centres and health posts were classified according to their initial status. We used a complete case analysis approach, except for the audit question concerning nasogastric tubes where the mode stratified by facility type and ownership was imputed (Supplementary material 2). This question was not asked the first day of data collection due to technical issues and was therefore missing. For the scores, the remaining missing values are imputed as 0. We performed a sensitivity analysis omitting the indicators with > 15% missing data (Supplementary material 2).

Data were described using counts, proportions, mean, and medians, and presented according to facility level, type of setting, and health care provider type where appropriate. We report 95% confidence intervals (CI) for the knowledge questionnaire, however not for the audit given the census approach used. We generated one audit score and one knowledge score as the primary outcomes of interest, based on guidelines [24,25], and consensus among authors, necessary for high-quality case management of cholera and other diarrhoeal diseases. Each question in the knowledge questionnaire was assigned a score of 0 (incorrect) or 1 (correct), or a fraction for those with multiple possible responses and respondents were only partially correct (e.g. 0.5 for selecting 2 correct and 1 incorrect of 4 options). These were summed and divided by the total number multiplied by 100, and the same process was repeated in each domain to provide domain-specific sub-scores. The intravenous score was excluded from the total knowledge score for comparisons as not all providers prescribed intravenous fluids. No audit score was calculated for drug shops since several indicators were not deemed relevant for a drug shop to meet. Questions used for creating scores are in Supplementary material 1.

Multivariable linear regression was performed to explore the association between the knowledge score (primary outcome) and key explanatory factors (facility type, cadre, age, setting, ownership, training within the previous 3 years, and province). One model was performed adjusting for all factors including providers working in health facilities, and a second was performed including all types of providers but excluding adjustment for facility type and ownership as these factors were not applicable for drug shop vendors and traditional health practitioners. Multivariable linear regression was performed for health facilities for the audit score (secondary outcome) and key explanatory factors (facility type, setting, ownership, external support within the previous 3 years, availability of a cholera treatment centre, and province). Audit sub-scores were compared through multiple logistic regression with a binary outcome indicating acceptable coverage (≥70%), given non-normal distributions for sub-scores. Proportions of individual indicators were compared with chi-square tests. A *p*-value < 0.05 was considered statistically significant. All quantitative analyses were performed using Stata version 16 (StataCorp LLC, College Station, Texas).

Qualitative and quantitative data were triangulated to explore common categories. Qualitative data were matched with quantitative categories, based on existing literature, and agreements and disagreements between data sources. Results for the audit are discussed for all facilities combined and drug shops, as well as for the following health facility strata: public hospitals; private hospitals; public health centres; and private health posts. No specific results are discussed for the remaining health facility strata as they had six or fewer facilities.

### Ethics

Written consent was obtained from all FGD participants and verbal consent was obtained for the audit and knowledge questionnaires. The provincial and health zone offices approved the study. Ethical approval was obtained from the ethical review board at Kinshasa School of Public Health (ESP/CE/16B/2022) and the Swedish Ethical Review Authority (Dnr 2022–02663–01).

## Results

**Figure 1.**
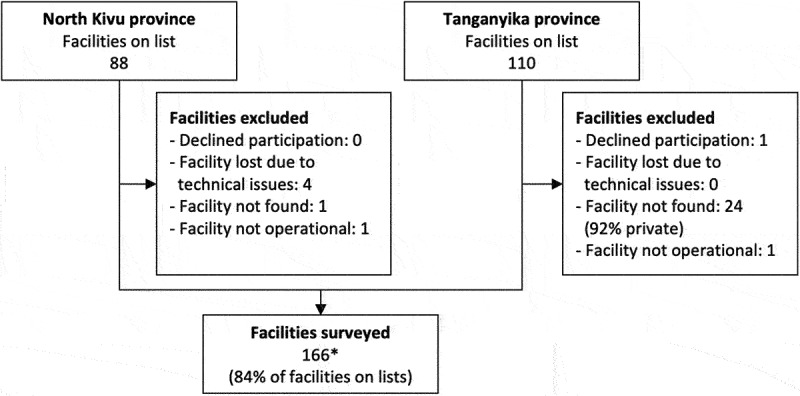
Health facilities included for quantiative data collection. *No audit questionnarie was registered for one facility in North Kivu.

We conducted 15 FGDs with 4–8 participants in each group (*n* = 84), 244 audits and 308 knowledge questionnaires (Table 1). In total 166/198 (84%) of health facilities were surveyed but for one health facility, no audit form was submitted and thus in total 165/198 were analysed. Most excluded health facilities were private facilities that could not be found (Figure 1). Of all the health facilities surveyed, 82/166 (49%) were in North Kivu, with 63/82 (77%) of these being urban. In Tanganyika, 67/84 (80%) of surveyed health facilities were urban. No knowledge questionnaire was registered for 4% (10/244) of facilities, and in one health facility, no audit form was submitted.

**Table 1.** Included facilities for audit questionnaire.

	Health facilities (*N* = 165)				
	Hospital (*n* = 82)	Health centre (*n* = 52)	Health post (*n* = 31)	Drug stores (*N* = 79)	
Public	26 (32)	44 (84)	6 (19)	0	
Private	50 (61)	4 (8)	24 (77)	79 (100)	
Confessional	6 (7)	4 (8)	1 (3)	0	
Included providers for knowledge questionnaire.					
	Health facility providers (*N* = 160)				
	Medical doctor (*n* = 16)	Nurse* (*n* = 136)	Other health facility cadre (*n* = 8)	Drug shop vendor (*N* = 75)	Traditional health practitioner (*N* = 73)
Public	4	64	5	0	0
Private	10	63	3	75	73
Confessional	2	8	0	0	0

We present qualitative and quantitative data together under the following categories: water sanitation and hygiene (WASH) and infection and prevention control (IPC); equipment, medication, and guidelines; case management; community engagement and cholera surveillance.

### WASH and IPC

Availability of water from an improved source on premises was reported for 76/165 (46%) of health facilities, and 134/165 (81%) had access to safe water infrastructure within 500 m. However, only 88/134 (66%) had water available during the visit. Water backup reservoirs of at least 500 L were available in 94/165 (57%) of health facilities (Table 2). A total of 66/163 (41%) of health facilities had water and soap available nearby the toilet, and 92/165 (56%) had water and soap available at the point of care. Having received external support in the three previous years was associated with a significantly higher audit IPC score (aOR 2.5; 95%CI, 0.8 to 7.3), however not for WASH (aOR 1.7; 95%CI, 0.8 to 3.6 [Supplementary material 2]). Improved in-facility WASH was not brought up during FGDs as a major priority for providers, however, focus on improved community WASH was mentioned as key to reduce the burden of cholera and other diarrhoeal diseases.

**Table 2.** Audit questionnaire results.

	Total		Public		Private		Confessional
	Health facilities (*N* = 165) (%)	Drug shop (*N* = 79) (%)	Hospital (*n* = 26) (%)	Health centre (*n* = 44) (%)	Hospital (*n* = 50) (%)	Health posts (*n* = 24) (%)	Hospital (*n* = 6) (%)
**WASH**							
Access to safe water source	134/165 (81)	58/79 (73)	22/26 (81)	34/44 (77)	42/50 (84)	19/24 (79)	5/6 (83)
Potable water available	90/165 (55)	36/79 (58)	16/26 (62)	23/44 (52)	24/50 (48)	12/24 (50)	4/6 (67)
Chlorine to prepare drinking water	85/165 (52)	40/79 (51)	16/26 (62)	24/44 (55)	26/50 (52)	9/24 (38)	5/6 (83)
Minimum 500 litre back-up reservoir	94/165 (57)	N/A	20/26 (77)	30/44 (68)	22/50 (56)	7/17 (24)	6/6 (100)
Adequate toilet available	162/165 (98)	N/A	26/26 (100)	44/44 (100)	49/50 (98)	23/24 (96)	6/6 (100)
Separate toilets for patients	100/163 (61)	N/A	20/26 (77)	34/44 (77)	29/50 (58)	5/23 (22)	5/6 (83)
Safe disposal of infectious body fluids	111/162 (69)	N/A	22/25 (88)	33/44 (75)	33/50 (66)	8/3 (25)	5/6 (83)
Safe disposal of soft waste	140/163 (86)	N/A	25/26 (96)	37/44 (84)	46/50 (95)	13/22 (59)	6/6 (100)
WASH score* (mean, SD)	**69 (21)**	**N/A**	**80 (15)**	**74 (20)**	**69 (17)**	**50 (22)**	**88 (19)**
**IPC**							
Water and soap nearby toilet	66/163 (41)	N/A	11/26 (42)	18/44 (41)	21/50 (42)	6/23 (26)	5/6 (83)
Water and soap at point of care	92/165 (56)	12/68 (18)	18/26 (69)	27/44 (62)	24/50 (48)	11/24 (46)	5/6 (83)
Chlorine for cleaning purposes	100/165 (61)	22/78 (28)	15/26 (58)	29/44 (66)	35/50 (70)	7/24 (29)	6/6 (100)
Availability of cleaning protocols	54/165 (33)	2/79 (3)	11/26 (42)	19/44 (43)	14/50 (28)	4/24 (17)	5/6 (83)
All cleaning personnel received training	51/165 (31)	N/A	13/26 (50)	18/44 (41)	14/50 (28)	1/23 (4)	4/6 (67)
Availability of person responsible for IPC	94/165 (57)	N/A	20/26 (77)	31/44 (70)	22/50 (44)	5/21 (21)	6/6 (100)
IPC score* (mean, SD)	**46 (29)**	**N/A**	**56 (25)**	**54 (28)**	**43 (27)**	**24 (21)**	**86 (27)**
**Cholera surveillance**							
At least one person trained in rapid response to cholera outbreak	37/165 (22)	N/A	7/26 (27)	11/44 (25)	10/50 (20)	6/24 (25)	0/6 (100)
At least one person can perform cholera line listing	123/165 (75)	N/A	20/26 (77)	35/44 (80)	34/50 (68)	18/24 (75)	3/6 (50)
Availability of cholera notification form	30/165 (18)	0/77 (0)	9/26 (35)	13/44 (30)	3/50 (6)	1/24 (4)	1/6 (17)
Cholera case definition on wall	62/165 (38)	1/78 (1)	15/26 (58)	26/44 (59)	10/50 (20)	2/24 (8)	3/3 (50)
Disease outbreak committee	39/165 (24)	N/A	9/26 (35)	18/44 (41)	5/50 (10)	0/24 (0)	2/6 (33)
Availability of internet or phone to transfer health data	97/165 (59)	10/78 (13)	17/26 (65)	33/44 (75)	25/50 (50)	8/24 (33)	5/6 (83)
Availability of personnel trained in contact tracing	19/165 (12)	N/A	5/26 (19)	5/44 (11)	4/50 (8)	4/24 (17)	0/6 (0)
Availability of RDT	16/165 (10)	0/79 (0)	7/26 (27)	8/44 (18)	0/50 (0)	1/24 (4)	0/6 (0)
Availability of transport medium for cholera test	24/165 (15)	N/A	9/26 (35)	10/44 (23)	3/50 (6)	0/24 (0)	2/6 (33)
Cholera surveillance score* (mean, SD)	**30 (22)**	**N/A**	**42 (24)**	**40 (25)**	**21 (14)**	**19 (15)**	**30 (23)**
**Guidelines**							
Cholera treatment guidelines	41/165 (25)	5/79 (6)	11/26 (38)	20/44(45)	7/50 (14)	1/24 (4)	2/6 (33)
Diarrhoea treatment guidelines	70/165 (42)	8/79 (10)	16/26 (62)	29/44 (66)	9/50 (18)	3/24 (13)	3/6 (50)
Dehydration guidelines	73/165 (44)	9/79 (11)	16/26 (62)	28/44 (64)	14/50 (28)	2/24 (8)	4/6 (67)
Guidelines score* (mean, SD)	**37 (39)**	**N/A**	**54 (41)**	**58 (39)**	**20 (32)**	**8 (18)**	**50 (35)**
**Community engagement**							
Staff specifically tasked with community sensitisation on cholera prevention and control	59/165 (36)	6/79 (8)	13/26 (50)	24/44 (55)	10/50 (20)	2/24 (8)	2/6 (33)
Staff specifically tasked with community sensitisation on childhood diarrhoea prevention and control	66/165 (40)	5/79 (6)	13/26 (46)	26/44 (59)	17/50 (34)	2/24 (8)	2/6 (33)
Organises community cholera sensitisation session	42/165 (25)	2/79 (3)	7/26 (27)	22/44 (50)	3/50 (6)	0/24 (0)	2/6 (33)
Organises community childhood diarrhoea sensitisation sessions	45/165 (27)	2/79 (3)	7/26 (27)	21/44 (48)	4/50 (8)	2/24 (8)	2/6 (33)
Engage community leaders and religious leaders to convey public health messages	72/165 (44)	2/79 (3)	13/26 (50)	31/44 (70)	12/50 (24)	3/24 (13)	2/6 (33)
Engage community volunteers to convey public health messages	80/165 (48)	3/79 (4)	15/26 (58)	35/44 (80)	15/50 (30)	4/24 (17)	3/6 (50)
Community engagement score* (mean, SD)	**37 (35)**	**N/A**	**43 (35)**	**60 (31)**	**20 (25)**	**9 (21)**	**36 (36)**
**Equipment**							
Can check electrolytes	21/165 (13)	N/A	7/26 (27)	2/44 (5)	8/50 (16)	0/24 (0)	2/6 (33)
Can check glucose	134/165 (81)	3/79 (4)	25/26 (96)	32/44 (73)	46/50 (92)	14/24 (58)	6/6 (100)
Functioning stethoscope	160/165 (97)	11/79 (14)	26/26 (100)	43/44 (98)	49/50 (98)	22/24 (90)	6/6 (100)
Functioning thermometer	154/165 (93)	30/79 (38)	25/26 (96)	40/44 (90)	47/50 (94)	24/24 (100)	6/6 (100)
Functioning scale	153/165 (93)	3/79 (4)	25/26 (96)	42/44 (96)	48/50 (96)	18/24) (75)	6/6 (100)
Availability of nasogastric tube	47/165 (28)	6/79 (8)	17/26 (65)	7/44 (16)	15/50 (30)	0/24 (0)	5/6 (83)
Equipment score* (mean, SD)	**68 (18)**	**N/A**	**80 (12)**	**63 (14)**	**71 (17)**	**54 (15)**	**86 (7)**
**Treatment**							
Availability of ORS	114/165 (69)	62/79 (78)	20/26 (77)	37/44 (84)	33/50 (66)	11/24 (46)	6/6 (100)
Availability of Zinc	95/165 (58)	44/79 (56)	17/26 (65)	31/44 (70)	26/50 (52)	8/24 (33)	6/6 (100)
Availability of Ringers lactate or NaCl	131/165 (79)	45/79 (57)	23/26 (88)	36/44 (82)	42/50 (84)	16/24 (67)	6/6 (100)
Availability of glucose 10% or 50%	109/165 (66)	30/79 (38)	20/26 (77)	28/44 (64)	34/50 (68)	11/24 (46)	6/6 (100)
Availability of ciprofloxacin	108/165 (65)	59/79 (75)	18/26 (69)	32/44 (73)	34/50 (68)	14/24 (58)	5/6 (83)
Treatment score* (mean, SD)	**68 (33)**	**N/A**	**75 (25)**	**75 (28)**	**68 (33)**	**50 (38)**	**97 (8)**
Total score*	**51 (17)**	**N/A**	**62 (13)**	**61 (12)**	**45 (14)**	**31 (11)**	**67 (17)**

Missing values are imputed as 0. *The score ranges from 0–100 and is calculated through dividing availability of each variable (equals 1 point) with the total number of variables in each category.

### Equipment, medication, and guidelines

Equipment to verify blood glucose concentrations was available in more than 90% of hospitals, more than 70% of health centres, and more than 50% of health posts (Table 2). Nasogastric tubes were found in 17/26 (65%) of public hospitals, 7/44 (16%) of public health centres, 15/50 (30%) of private hospitals but in none of the 24 private health posts. ORS was available in close to 80% of public hospitals, public health centres and drug shops compared to close to 50% of private hospitals and private health posts. Zinc availability was lower, at all types of facilities, compared to ORS (range −12 to −22% lower, Table 2). A ten-day treatment with zinc was twice as expensive compared to 1 L of ORS (906 versus 490 Congolese Franc [0.4 and 0.2 USD)) and profit was 17% higher (Supplementary material 2). Availability of intravenous fluids – Ringer’s lactate or NaCl, was between 2% and 18% higher than for ORS for all types of health facilities, except drug shops, where it was 21% lower. Stockouts were reported in 24/244 (10%) facilities for ORS, 32/244 (13%) for zinc and 8/244 (3%) for intravenous fluids. During FGDs, providers mentioned that lack of materials and medications limited their capacity to provide high-quality care.

Guidelines for diarrhoeal disease case management were mentioned to be used by most health facility personnel during FGDs. However, availability of guidelines was only found in 70/165 (42%) of all health facilities and was 37% lower (*p* < 0.001) for private facilities compared to public facilities. The availability of cholera treatment guidelines was 19% lower (*p* = 0.001) than childhood diarrhoea guidelines for all health facilities combined. Guideline differences between the government and NGOs was brought up as a complicating factor during the FGDs. Some drug shop vendors acknowledge basing treatment suggestions on protocols however, this did not come up during the FGDs with traditional health practitioners. Medical doctors, nurses and drug shop vendors asked for a clear protocol for providers to improve case management, with guidelines attributed to facilitating treatment among the providers that were already using them.

The procedures to be followed for each plan are contained in the protocol that we follow. This protocol is like a bible for us providers. It is these three plans that help us to know which medicine to give the child according to the [dehydration] signs. (Nurse, Kirothse)

**Figure 2.**
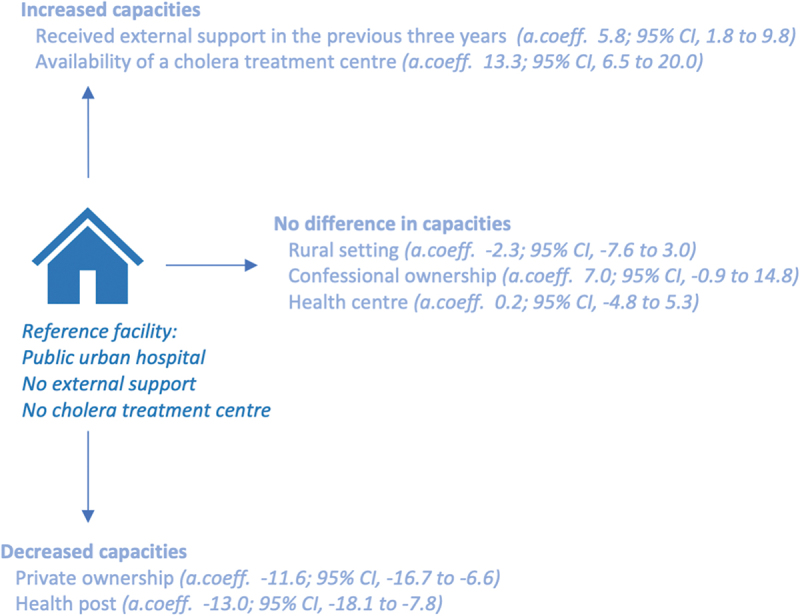
Presentation of adjusted linear regression coefficient for the total audit score. Adjusted for health facility type, ownership, setting, external support in the previous three years, availability of cholera treatment centre, and province.

When exploring audit scores, private facilities had a −11.6 (95% CI, −16.7 to −6.6) lower adjusted mean total audit score compared to public facilities (Figure 2). No significant difference was found between the urban and the rural setting. Having received any kind of external support in the three previous years was associated with a 5.8-point (95% CI, 1.8 to 9.8) higher adjusted mean audit score. Cholera treatment centres had a 13.3 (95% CI; 6.5 to 20.0) point higher adjusted mean score.

For the audit sub-scores (analysed with logistic regression) significantly fewer private than public facilities reached the ≥ 70% score for WASH (aOR 0.1.; 95%CI, 0.0 to 0.4), guidelines (aOR 0.1; 95%CI, 0.0 to 0.5), and equipment (aOR 0.4; 95%CI, 0.1 to 1.0) (Supplementary material 2). Significantly fewer rural compared to urban facilities met the criterium for WASH (aOR 0.2; 95%CI, 0.1 to 0.7), and IPC (aOR 0.1; 95%CI, 0.0 to 0.8). No private (0/78) or confessional (0/11) facilities met the 70% threshold for the cholera surveillance score compared to 9/76 for public facilities.

#### Case management

Overall, 124/160 (78%) of medical personnel, 38/75 (51%) of drug shop vendors and 4/71 (6%) of traditional health practitioners reported to have prescribed both ORS and zinc to the last child they treated with diarrhoea. When considering only ORS, the prescription was 9%, 33%, and 15% higher respectively. Of the providers that reported prescribing homemade ORS in the previous 6 months several could not explain the correct formulation: 5/15 (33%) of health facility personnel, 2/13 (15%) of drug shop vendors, and 10/21 (48%) of traditional health practitioners. Intravenous fluid was reported to have been prescribed by 51/160 (32%) of health facility personnel to the last child with diarrhoea. Antibiotics were prescribed in 74/160 (46%) of diarrhoea cases for health facilities, in 44/75 (59%) for drug shops, and 7/73 (10%) for traditional health practitioners. When asked when antibiotics should be prescribed the correct indication was identified in 25% of cases for health facility personnel, 26% for drug shop vendors, and 13% for traditional health practitioners (Table 3).

**Table 3.** Knowledge questionnaire results.

	Health facility personnel (*n* = 160) (%; 95% CI)	Drug shop vendor (*n* = 75) (%; 95% CI)	Traditional health practitioner (*n* = 73) (%; 95% CI)
**General diarrhoea knowledge**			
Knows definition of acute water diarrhoea	99/160 (62; 54–69)	31/74 (42; 31–54)	25/73 (34; 24–46)
Knows treatment for uncomplicated diarrhoea	57/160 (36; 29–43)	8/75 (11; 5–20)	0/72 (0; N/A)
Can explain what ORS is	121/160 (76; 68–82)	55/75 (73; 62–82)	31/72 (43; 32–55)
Can explain how to prepare ORS	137/160 (86; 79–90)	62/75 (83; 72–90)	37/72 (51; 40–63)
General diarrhoea knowledge score*	**65 (95% CI: 61–69)**	**52 (95% CI: 46–58)**	**32 (95% CI: 27–37)**
**Dehydration classification**			
Can identify moderate dehydration	73/160 (46; 38–52)	19/72 (26; 17–38)	15/72 (21; 13–32)
Knows clinical dehydration signs** *(mean, 95% CI, N)*	58 (54–61; 160)	41 (37–46; 74)	34 (30–40; 73)
Dehydration classification score*	**52 (95% CI: 47–56)**	**33 (95% CI: 27–39)**	**28 (95% CI: 22–33)**
**Correct treatment**			
Prescribed ORS and zinc to last child with diarrhoea	124/160 (78; 70–83)	38/75 (51; 39–62)	4/71 (6; 2–14)
Knows main priority with diarrhoea treatment (prevent dehydration)	99/160 (62; 54–69)	36/74 (49; 37–60)	21/73 (29; 19–40)
Knows indication for antibiotics** *(mean, 95% CI, N)*	25 (20–30; 158)	26 (18–34; 72)	13 (6–19; 69)
Knows treatment plan A	75/156 (48; 40–56)	18/73 (25; 16–36)	9/70 (13; 7–23)
Knows indication for additional ORS	119/160 (74; 67–81)	45/74 (61; 49–71)	26/72 (36; 26–48)
Knows stage of dehydration to use Plan B	89/160 (56; 48–63)	24/74 (32; 23–44)	6/73 (8; 4–17)
Is aware of the importance to re-evaluate the child	131/160 (82; 75–87)	44/74 (59; 48–70)	27/73 (37; 27–49)
Can identify when nasogastric tube is preferred	21/159 (13; 9–19)	3/71 (4; 1–13)	2/66 (3; 1–12)
Knows the basal fluid need for a 3-year-old 12 kg child	21/151 (14; 9–20)	3/75 (4; 1–12)	3/70 (4; 1–13)
Knows indication to verify glucose level	10/160 (6; 3–11)	1/67 (1; 0–10)	0/68 (0; N/A)
Correct treatment score*	**45 (95% CI: 44–47)**	**31 (95% CI: 27–34)**	**15 (95% CI: 12–18)**
**Advice to caregivers**			
Correct advice regarding breastfeeding	156/159 (98; 94–99)	58/74 (78; 67–86)	62/71 (87; 77–93)
Correct advice regarding food-intake	102/160 (64; 56–71)	47/75 (63; 51–73)	40/72 (56; 44–67)
Correct advice regarding fluid intake	128/160 (80; 73–86)	47/75 (63; 51–73)	33/73 (45; 34–57)
Correct advice when to return to facility** *(mean, 95% CI, N)*	67 (62–71; 157)	55 (48–63; 73)	41 (35–47; 71)
Advice score*	**77 (95% CI: 74–79)**	**64 (95% CI: 59–70)**	**56 (95% CI: 51–61)**
Intravenous fluids***			
Knows WHO clinical definition of shock** *(mean, 95% CI, N)*	50 (44–56; 144)	58 (38–79; 9)	0 (N/A; 3)
Knows treatment plan C	59/138 (43; 35–51)	5/10 (50; 19–81)	0/3 (0; N/A)
Knows treatment of shock	44/141 (31; 24–39)	3/10 (30; 8–67)	0/3 (0; N/A)
Knows that IV-fluids should contain electrolytes	115/143 (80; 73–86)	8/10 (80; 40–96)	0/3 (0; N/A)
Intravenous fluid score*	**50 (95% CI: 46–54)**	**53 (95% CI: 34–72)**	**0 (95% CI: N/A)**
**Prevention**			
Knows how to prevent and/or protect** *(mean, 95% CI, N)*	70 (67–74; 160)	65 (60–71; 75)	59 (53–65; 73)
Knows in what clinical situations to wash hands** *(mean, 95% CI, N)*	51 (48–54; 160)	33 (28–38; 75)	37 (33–40; 73)
Prevention score*	**61 (95% CI: 58–63)**	**49 (95% CI: 45–53)**	**48 (95% CI: 44–52)**
**Cholera**			
Knows that vaccinations effect reduces with time	100/159 (63; 55–70)	40/74 (53, 41–64)	42/73 (58; 46–68)
Knows definition of suspected cholera case during an outbreak	86/151 (58; 50–65)	28/68 (41, 30–53)	15/73 (21; 13–32)
Knows definition of suspected cholera case when no outbreak	71/156 (46; 38–53)	32/67 (48, 36–60)	36/72 (50; 38–62)
Cholera score*	**54 (95% CI: 50–58)**	**44 (95% CI: 38–50)**	**42 (95% CI: 36–49)**
**Total score**	**59 (95% CI: 57–60)**	**46 (95% CI: 43–48)**	**37 (95% CI: 34–39)**

Missing values are imputed as 0. *The score ranges from 0–100 and is calculated through dividing of the sum of all correct answers (1 point per correct answer) with the total number of questions in each category. **Multiple choice question, not possible to present as a n/N since fraction scores, between 0–1, was possible. *** Not included in total score due to that not all providers prescribe intravenous fluids.

It was mentioned during FGDs that caregivers often do not perceive ORS as a valid treatment wanting intravenous fluids instead. Conversely, some caregivers were said to be afraid of intravenous treatment.

*[For] Moderate dehydration, we advise to give ORS only after liquid stools or when the child wants, but the habits in the context we are working will make us prescribe Ringer’s lactate [intravenous fluid] even though it is not indicated in the protocol.* (Nurse, Karisimbi)

The mean total knowledge score was 59 (95% CI: 57 to 60) for health facility personnel, 46 (95% CI: 43 to 48) for drug shop vendors and 37 (95% CI: 34 to 39) for traditional health practitioners. The treatment score had the lowest overall score of all sub-scores (Table 3). A total of 69/160 (43%) of health facility personnel, 20/75 (27%) of drug shop vendors, and 36/73 (49%) of traditional health practitioners did not consider themselves to have sufficient training in diarrhoea case management. The best performing sub-category was advice to caregivers; and the specific questions with the lowest percentage of correct answers were when to test for hypoglycaemia, indication for use of nasogastric tube, and maintenance fluid requirements based on weight, all related to treatment. Concerning nasogastric tubes, one nurse mentioned during the FGD that this was occasionally used when vein access was not possible. However, providing treatment through a nasogastric tube was not always accepted by the caregiver, due to fear that the nasogastric tube would hurt the child.

‘*We can even rehydrate from a tube if there is a difficulty drinking, but when you place a tube, the family will perceive that you are in the process of pressing in [French translation ‘enfoncer’], of killing the patient*.‘ (Medical doctor, Kirotshe)

**Figure 3.**
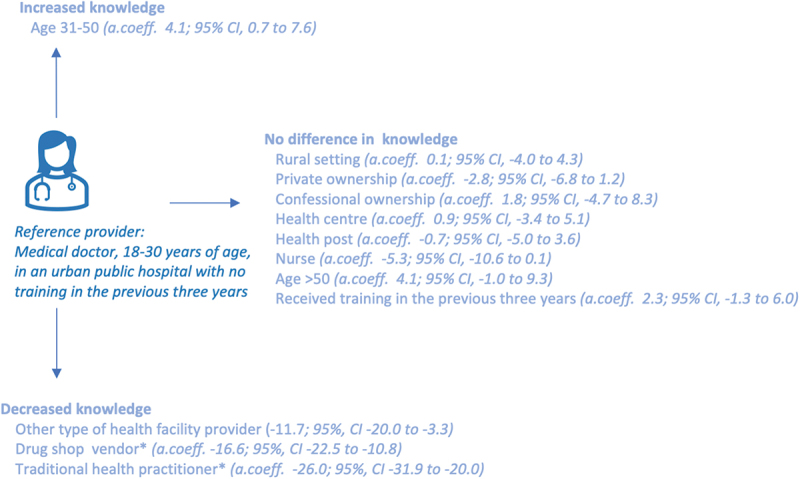
Presentation of adjusted linear regression coefficient for the total knowledge score. Adjusted for health facility type, cadre, ownership, setting, age, training in the previous three years, and province. * The adjusted coefficient for drug shop vendors and traditional health practitioner is not adjusted for health facility type and ownership.

Medical doctors had a higher total knowledge score than nurses (a.coeff −5.3; 95% CI −10.6 to 0.1), and significantly higher knowledge score than other types of health facility providers (a.coeff −11.7; 95% CI, −20.0 to −3.3, [Figure 3]). No difference in knowledge score was observed between rural or urban settings; different types of ownership; nor for having received training during the previous 3 years. For the adjusted regression including drug shop vendors and traditional health practitioners, a −16.6 (95% CI, −22.5 to −10.8) point lower adjusted mean knowledge score for drug shop vendors and a −26.0 (95% CI, −31.9 to −20.0) lower adjusted mean score for traditional health practitioners compared to medical doctors was found.

#### Community engagement

Performing community sensitisation was most common for public health centres where 22/44 (50%) did it for cholera and 21/44 (48%) for childhood diarrhoea, followed by public hospitals 7/26 (27%). Significantly fewer private health facilities performed community sensitisation (aOR 0.2; 95%CI, 0.1 to 0.8). All three provider groups brought up in the FGDs that educating the community about protection, prevention, and home treatment measures, and seeking care early is key to lowering diarrhoea and cholera case burdens. While educating caregivers and communities about preventive practices was said to be part of their current practice, they emphasised that they could do this more. When probed, several participants said that it would be a good idea to work more interactively with the community and to make better use of the expertise the providers have. Health facility personnel emphasised that qualified health personnel, and persons with great influence in the community, should be used to educate the community, which was not always the case today. Several traditional health practitioners wished to get training to perform similar services as community health care workers and assist with sensitisation. At the same time, providers were clear that, as long as the underlying problems are not solved, sensitisation will have little effect, by exemplifying how they themselves had problem getting sufficient water and that prevention methods, as buying chlorine tablets, was down prioritised by caregivers due to costs with caregivers prioritising other expenses.

*Even if you told me to wash my hands, how would I do it? I buy a 500 FC [local currency] bucket. Will I end up washing my hands? No. I economize to save my water, I have to wash the dishes in small basins, so that I do not run out of water.*

(Medical doctor, Karismbi)

### Cholera surveillance

A cholera case definition displayed on the wall was available in 62/165 (38%) of health facilities, with 43% (*p = 0.001)* lower availability in private facilities compared to public. Availability was 1/78 (1%) for drug shops. A total of 65/165 (40%) of health facilities had cholera definition available somewhere in the facility. Furthermore, 16/165 (10%) of health facilities had access to cholera rapid diagnostic tests, and availability of transport mediums for cholera samples ranged between 0% and 35% for the different health facility strata.

For cholera recognition, rice water stool was the most common reason given to suspect cholera from all types of providers 262/292 (90%), and repeated abundant liquid stools 90/292 (31%) – the community definition from the national cholera program, was the second most common. Knowledge about case definitions for cholera, both during an outbreak and in the absence of an outbreak, was similar between groups (range 41–58%) except for traditional health practitioners concerning the definition during an outbreak where 15/73 (21%) knew the case definition. Regarding identifying cases, during FGDs drug shop vendors mentioned treating cholera to be illegal and that they did not see any cholera cases in the drug shop. Concurrently drug shop vendors brought up the perspective that the community cannot separate cholera from other diarrhoeal disease and, thus, the importance of training drug shop vendors to recognise cholera for referral. Traditional health practitioners mentioned treating cholera informally and that their plant-based medications were effective. Traditional health practitioners also brought up that they try to help refer cholera cases to health facilities.

*… few are those who know [how to] differentiate between simple diarrhoea and cholera. For us Congolese when the child makes [loose] stools the first thing we do is [to] resort to the drug shop [rather] than to the hospital. Many have this culture, and that said, during cholera outbreaks we also play a big role.*

(Drug shop vendor, urban Kalemie)

## Discussion

In this mixed methods study exploring capacity for case management of diarrhoeal disease and cholera surveillance in cholera ‘hotspots,’ we found weak facility infrastructure and knowledge about case management. Unsurprisingly, knowledge was significantly lower among drug shop vendors and traditional health practitioners compared to medical doctors and nurses. Audit results found that private facilities were more poorly equipped than public facilities, however, provider knowledge was not significantly different. Additionally, our data indicate that all three main healthcare provider groups in this setting are poorly prepared for cholera surveillance.

WASH and IPC indicators were low for health facilities. Especially noteworthy was only half of facilities having access to a safe water source actually had water available at the time of the survey. Access to safe drinking water is a prerequisite to prepare ORS and availability of water a necessity to maintain IPC measures [31], and needs further attention. Overall, private facilities had lower audit scores. Data from 2017 indicate that private providers are important for diarrhoea care with 27% of caregivers in North Kivu and 12% in Tanganyika seeking care in private health facilities when their children have diarrhoea, compared to 36% and 7%, respectively, for public providers [8]. Interestingly, on a national level in the DRC, private facilities have been reported to have better infrastructure [32,33]. We found that external support was mainly concentrated to public facilities, and with cholera hotspots receiving larger focus from external actors than many other parts of the country, which may explain this finding. A system of support, supervision, governance, and accountability to ensure all facilities follow minimal standards could improve the situation. However, facilities are currently poorly regulated and supervised in the DRC, partly given the limited fiscal space for the health authorities [34].

The availability and prescription of ORS was relatively high compared to zinc, especially in drug shops. It is important to note that our numbers are considerably higher than the latest Multiple Indicator Cluster Survey for ORS treatment of diarrhoea in children [8]. The latter reports received treatment, not prescribed, and includes children who are never taken for care by a provider, which likely helps explain the difference. Nonetheless, what treatment providers prescribe and promote is an important mediator to improve use and coverage. Increasing use of ORS and zinc for diarrhoeal diseases has been shown to be possible by targeting demand and supply barriers, there among pricing, product quality, provider dispensing practices, stocking rates, and consumer demands [35]. Our price data showed that one full course of zinc treatment was about twice as expensive but with a profit similar to 1 L of ORS. Several litres of ORS are often needed to be prescribed; hence, our price data suggest that the financial incentive for prioritising ORS is higher both for the provider and the caregiver. Subsidisation of zinc treatment could therefore potentially be of value to increase coverage. Treatment according to guidelines was furthermore found to not always be accepted by caregivers. Further research exploring how providers, and caregivers, decide on treatment in the current study setting would be of value to inform policies that nudge providers to recommend and the community to demand ORS and zinc for treating diarrhoeal diseases.

We found low knowledge scores for all types of providers. This is consistent with a 2022 study from Lubumbashi, DRC, which found low knowledge about cholera signs, prevention, and treatment among health care workers and community leaders [36]. Providers in our study performed particularly poorly concerning dosing schedules, calculation of fluid needs, use of the nasogastric tube, and when to check for low blood sugar which have low cost and lifesaving potential [37]. For timely improvement of case management there is a need for opportunities for continued professional development of existing providers and at the same time improving the quality of basic training [38]. Previous research suggests a modest effect on knowledge of isolated training [39], and in our analysis, no difference was found for knowledge scores between providers that had and had not received training in the previous 3 years. Combining training with the establishment of an in-house culture of follow-up and a stimulating learning environment, as acknowledged by the DRC Ministry of Health [34], and in addition improving supervision would likely improve knowledge and practices [39].

The low knowledge scores found for drug shop vendors and traditional health practitioners is unsurprising, albeit the number of drug shops in the DRC are numerous [40], and previous research demonstrates that they, together with traditional health practitioners, are important providers of care [12,41,42], which our study also found support for. Including them in health policies could potentially reduce the risk of low-quality diarrhoea case management and would concurrently require strategies to prevent potential negative side effects (e.g. indirect legitimation of non-approved practices). Drug shops and traditional health practitioners are under the Ministry of Health; however, they are currently poorly regulated [43–45].

Our analysis indicates that all three provider groups are poorly prepared and poorly integrated in cholera surveillance. Few facilities had available definitions and guidelines describing what to do with a suspected cholera case. Additionally, knowledge about case definitions, correct management, and prevention measures was low. Concurrently providers acknowledge that they likely treat cholera due to the difficulty to distinguish between cholera and other diarrhoeal diseases. A larger focus on ensuring that providers are aware of case definitions and procedures for suspected cholera cases would likely be beneficial. This has support by GTFCC guidelines [9]. Development of coherent guidelines, with clear definitions of cases that are clearly displayed in facilities, that explain what measures to take when a suspected case of cholera is identified by a provider if implemented jointly with training could likely improve integration of facilities and providers in cholera surveillance.

This study had several limitations. First, our study was performed in easy access areas in both provinces. Harder to reach areas in the same provinces would likely have worse results. We would also like to underscore that we only included one provider per facility for the knowledge questionnaire among all cadres providing diarrhoea case management independently. This means that we have not studied the best available knowledge, but rather the average knowledge available in the facility. Second, we did not include caregivers or prayer homes which are important providers of care in the DRC. These perspectives were being elicited from concurrent research by UNICEF Integrated Analytics Cell [18], and we therefore did not want to duplicate efforts. Third, questionnaires had many questions which risk introducing respondent fatigue. However, we did not find any patterns in the data to indicate that questions later in the questionnaire had worse performance or higher missing data. Fourth, some questions had high percentage of missing data and were imputed as zero when calculating the scores. Our sensitivity analysis explored this approach and did not result in any significant changes except that the WASH sub-score was not any longer significantly different for private nor rural facilities. Fifth, FGD moderators and data collectors were employed by UNICEF. UNICEF engages actively in cholera and diarrhoeal diseases response in the settings where the research was conducted which can have affected how responses to posed questions were given. However, interviewers were clear about the anonymity of study participants as well as the importance of obtaining truthful answers to questions to be able to inform future interventions. Concurrently we want to highlight that this study contributes important aspects to wider healthcare systems strengthening in outbreak settings that today is recommended but rarely studied. The findings in this study may well be generalisable to other cholera hotspots, given the general tendency of vertical programmes to limit cholera and that cholera outbreaks predominantly occur in the most fragile settings where pluralistic health systems are common.

## Conclusions

We identified three specific areas in need of attention. First, increasing availability and awareness of case definitions and procedures for suspected cholera cases to improve cholera surveillance and control. Case definitions for cholera were available in few facilities, and only about half of the providers knew when to suspect cholera. Second, increased support and supervision to ensure that facilities are adequately equipped to provide safe care, with a particular focus on private providers. WASH and IPC health facility infrastructure was concerningly poor, with private facilities performing worse than public. Third, emphasising more nuanced aspects of case management such as dosing schedules, fluid needs, use of nasogastric tubes, and when to suspect hypoglycaemia in future opportunities given to providers for continued professional development since these were the areas where providers had the lowest knowledge.

## Supplementary Material

Supplementary material 1 DRC Diarrhoea GHA cleaned.docx

Supplementary material 2 DRC Diarrhoea GHA_.docx

## Data Availability

The data underlying this article were provided by UNICEF CAI by permission. Data can be shared on request, for the purposes of research only, depending on permission from UNICEF CAI. Requests should be sent to scarter@unicef.org.

## Paper context

Wider healthcare-strengthening interventions are recommended in cholera hotspots to improve preparedness for future outbreaks, and our data highlights clear gaps in this approach in a high-burden setting. We identified poor health facility water sanitation and hygiene measures, and large knowledge gaps in calculating fluid requirements, identifying hypoglycaemia, and cholera case definitions. More focus on these gaps could also have synergies with other types of diarrhoeal diseases that contribute greater mortality than cholera.
