# Supplementary material for: Facility capacity and provider knowledge for cholera surveillance and diarrhoea case management in cholera hotspots in the Democratic Republic of Congo – a mixed-methods study
Source: Glob Health Action. 2024 Mar 5;17(1):2317774. doi: 10.1080/16549716.2024.2317774 (PMC10916892; doi:10.1080/16549716.2024.2317774)
Supplement: Supplementary material 1 DRC Diarrhoea GHA cleaned.docx [file ZGHA_A_2317774_SM2508.docx]

**Supplementary material 1**

**Supplement Table 1.** Intended sampling.

|  | Health zones | | | |  |  |
| --- | --- | --- | --- | --- | --- | --- |
| Focus group discussions | Karismibi | Kirotshe | Kalemie urban | Kalemie rural | Sampling approach | Comment |
| Total | 4 | 4 | 4 | 3 |  | Most distant and insecure areas excluded from study area. |
| Medical doctors | 1 | 1 | 1 | 0 | Purposefully selection of health facilities from recently updated lists, provided by the provincial health offices, to represent as great heterogenicity as possible for level of care, ownership, and geographical position. Doctors available on the day of the discussion were selected. | No focus group was planned for medical doctors in rural Kalemie because facilities in this area are spread over large distances and only have one or two doctors employed, making the performance of FGDs unethical since this would leave the facilities without doctors for an extended period. |
| Nurses | 1 | 1 | 1 | 1 | Same as for medical doctors. |  |
| Drug shop vendors | 1 | 1 | 1 | 1 | Pharmacies and drug shops were identified from lists provided by the provincial health offices except for rural Kalemie where no such list existed. For this setting, the research team recruited participants from four drug shops of different standards operating in a limited geographical zone to facilitate participation. | No difference was made between registered pharmacies and informal drug shops. In 2017 there were 109 pharmacies and an estimated 8 000 – 10 000 informal drug shops in the DRC^.1^ |
| Traditional health practitioners | 1 | 1 | 1 | 1 | Purposefully selected with help from the traditional health practitioner’s association to represent different health areas within the health zone. | All types of traditional health practitioners registered by the local traditional health practitioner’s association were included. |
| Quantitative data |  |  |  |  |  |  |
| Health facilities | Census | Census | Census | Census | Recently updated lists of known health facilities from provincial health office. | Recommended by established guidelines (due to low number of facilities).^2^ |
| Drug shops | 20 | 20 | 20 | 20 | We intended to randomly select but given pragmatic limitations employed a convenience approach selecting the second closest pharmacy and/or drug shop with a 200 m minimum distance from a randomly selected health facility, stratified by health area. | We used a broad definition of pharmacies where all stores that specialised in selling medications were included meaning both registered pharmacies and informal drug shops. In 2017 there were 109 pharmacies and an estimated 8 000 – 10 000 informal drug shops in the DRC.^1^  A distance of 200m was chosen to minimise risk of including health facilities that work closely with the health facility. |
| Traditional health practitioners | 20 | 20 | 20 | 20 | Same as for focus group discussion. | No audit performed for traditional health practitioners. All types of traditional health practitioners registered by the local traditional health practitioner’s association were included. |

**Supplement Table 2.** Audit questionnaire. Included categories and specific question for each category. Each correct answer gives one point. A score is generated for each category through dividing total point for category by number of questions for the categories x 100. All category scores are then summed together and divided by the total number of categories for a total mean score for each facility.

| Category | Question | Answers | Score coding | Comment |
| --- | --- | --- | --- | --- |
| WASH | What is the main water supply for the facility? | 1. Piped supply inside the building  2. Piped supply outside the building  3. Public tap/standpipe  4. Tube well/borehole  5. Protected well  6. Unprotected well  7. Protected spring  8. Unprotected spring  9. Rainwater catchment  10. Bottled water  11.Water vendor  12.Tanker truck  13.No water source | (1 or 2 or 3 or 4 or 5 or 7 or 9 or 10 or 12) & within 500 m from facility premises = 1 point | Does not contain surface water specifically |
|  | Do you have potable water currently available? | 0. No  1. Yes, normally, but not today  2. Yes, same as for facility  3. Yes, water bottles  4. Yes boiled water  5. Yes water treated with tablets or chlorine | (2+question above) or 3 or 4 or 5 = 1 point |  |
|  | Do you have a product to chlorinate water (for drinking) currently available? | 0.No  1.Yes, liquid chlorine  2.Yes, Aquatabs  3.Yes, Sachet Pur | 1 or 2 or 3 = 1 point |  |
|  | Total volume of reservoirs to conserve water (in litres): | Integer | >500 = 1 point |  |
|  | What type of toilet/latrines are at the facility for patients? | 1. Flush/pour-flush toilet to sewer connection  2. Flush/pour-flush toilet to septic tank  3. Improved pit latrine  4. Pit latrine with slab  5. Composting toilet  6. Bucket  7. Hanging toilet/latrine  8. Bush  9. Lake/river  10. No toilet/latrine | 1 or 2 or 3 or 4 or 5 = 1 point |  |
|  | Separate toilets for patients and personnel? | 0.No  1.Yes | 1 = 1 point |  |
|  | How does this facility usually treat/dispose of infectious liquids as vomit and faeces? | 1. Not treated, dumped in latrine  2. Treated with chlorine then dumped in latrine  3. Not treated, but buried in lined, protected pit  4. Treated with chlorine then dumped in protected pit  5. Not treated, but collected for medical waste disposal off-site  6. Treated and collected for medical waste disposal  7. Open dumping without treatment | 2 or 4 or 6 = 1 point |  |
|  | How does this facility usually treat/dispose of infectious soft waste? | 1. Autoclaved  2. Incinerated (two chamber 850-1000 °C)  3. Incinerated (other)  4. Burning in a protected pit  5. Not treated, but buried in lined, protected pit  6. Not treated, but collected for medical waste disposal off-site  7. Open dumping without treatment  8. Open burning | 1 or 2 or 3 or 4 or 5 or 6 = 1 point |  |
| IPC | Are there hand hygiene facilities available at toilets? | 0. No  1. Water only  2. Soap + water  3. Alcohol gel/rub only  4. Soap + water & alcohol | 2 or 4 = 1 point | Since diarrhoeal diseases alcohol gel considered insufficient |
|  | Are there hand hygiene facilities at point of care? | 0. No  1. Water only  2. Soap + water  3. Alcohol gel/rub only  4. Soap + water & alcohol | 2 or 4 = 1 point | Since diarrhoeal diseases alcohol gel considered insufficient  The most frequently used clinical room was chosen |
|  | Do you have chlorine available for cleaning? | 0. No  1. Yes, observed  2. Yes, reported | 1 or 2 = 1 point | Both reported and observed included |
|  | Are cleaning protocols available? | 0. No  1. Yes, observed  2. Yes, reported | 1 or 2 = 1 point | Both reported and observed included |
|  | Have all staff responsible for cleaning received training? | 0. No, none have been trained  1. Yes, all have been trained  2. No, some but not all have been trained  3. No, there is no staff responsible for cleaning | 1 = 1 point |  |
|  | Is there an IPC focal person at the health facility? | 0. No  1. Yes | 1 = 1 point |  |
| Surveillance | Do you have staff trained in rapid response to cholera outbreak, who has trained them? | 0. No  1. Yes – by MoH programme  2. Yes – by NGO programme  3. Yes – by other (specify) | 1 or 2 or 3 = 1 point |  |
|  | How many staff can perform epidemiological reporting of cholera (i.e. line listing)? | 0.0  1.1  1.2  1.3 a 4  1.>4  5. Everyone | 1 or 2 or 3 or 4 or 5 = 1 point |  |
|  | Do you have a document about investigation of cholera cases in the community? | 0. No  1. Yes, observed  2. Yes, reported | 1 or 2 = 1 point | Both reported and observed included |
|  | Do you have a disease outbreak preparedness committee, if yes when did it last meet? | 0. No  1. Yes, last 3 months  2. Yes, last 12 months  3. Yes, over 12 months ago | 1 or 2 or 3 = 1 point | Not considering last time met |
|  | Is there a case definition of suspected cholera on the wall (including signage for cholera symptoms)? | 0. No  1. Yes, reported but not seen  2. Yes, observed  3. Observed, definition on the wall but not mentioning cholera symptoms  4. A printed version exist in the facility, but it is not attached to the wall | 1 or 2 or 3 = 1 point | Not on wall counted as 0 |
|  | Does your health facility have internet to facilitate timely reporting of surveillance data to the relevant institutions? | 0. No  1.Yes  2. Only telephone for calls/sms | 1 or 2 = 1 point | Internet or phone counted as 1 point. |
|  | Do you have staff trained in contact tracing of cholera cases and their close contacts? | 0. No  1. Yes  2. No, but for other diseases | 1 = 1 point |  |
|  | Does the facility have functioning tools to diagnose cholera available? | 0. No  1. Yes, rapid-diagnostic test, observed and not expired  2. Yes, rapid-diagnostic test, only reported or expired  3. Yes, lab-verified testing (Culture or PCR) | 1 or 2 = 1 point | Only gives point for RDT |
|  | Does the health facility have transport media for cholera diagnosis available? | 0. No  1. Yes, filter paper  2. Yes, cary blair medium  3. Yes, locally developed medium  4. Yes, other, specify | 1 or 2 or 3= 1 Point | Includes locally development medium even tough might not be according to standard |
| Guidelines | Do you have cholera treatment guidelines? | 0. No  1. Yes, reported but not seen  2. Yes, observed | 1 or 2 = 1 point | Both reported and observed included |
|  | Do you have childhood diarrhoea treatment guidelines? | 0. No  1. Yes, reported but not seen  2. Yes, observed | 1 or 2 = 1 point | Both reported and observed included |
|  | Do you have guidelines for assessing dehydration in children? | 0. No  1. Yes, reported but not seen  2. Yes, observed | 1 or 2 = 1 point | Both reported and observed included |
| Community engagement | Are there staff specifically tasked with community mobilisation/sensitisation on cholera prevention and control? | 0. No  1. Yes | 1= 1 point |  |
|  | Are there staff specifically tasked with community mobilisation/sensitisation on childhood diarrhoea prevention and control? | 0. No  1. Yes | 1= 1 point |  |
|  | Does health facility organise cholera awareness sessions in community gatherings (e.g. schools and religious centres) | 0. No  1. Yes, last 3 months  2. Yes, last 12 months  3. Yes, over 12 months ago | 1 or 2 or 3 = 1 point | Not considering last time met |
|  | Does health facility organise awareness sessions in community gatherings (e.g. schools and religious centres) concerning childhood diarrhoea? | 0. No  1. Yes, last 3 months  2. Yes, last 12 months  3. Yes, over 12 months ago | 1 or 2 or 3 = 1 point | Not considering last time met |
|  | Does health facility regularly engage community leaders and religious leaders to convey key public health messages in preparedness for an outbreak? | 0. No  1. Yes, last 3 months  2. Yes, last 12 months  3. Yes, over 12 months ago | 1 or 2 or 3 = 1 point | Not considering last time met |
|  | Des health facility often engages community volunteers to promote health awareness? | 0. No  1. Yes, last 3 months  2. Yes, last 12 months  3. Yes, over 12 months ago | 1 or 2 or 3 = 1 point | Not considering last time met |
| Equipment | Do you have functioning technology to check electrolytes? | 0. Non  1. Yes , point of care test (e.g., blood gas machine)  2. Yes, laboratory testing | 1 or 2 = 1 point |  |
|  | Do you have functioning technology to check blood glucose? | 0. No  1. Yes, point of care test (e.g., a glucometer), with strips available  2. Yes, laboratory testing | 1 or 2 = 1 point |  |
|  | Do you have a stethoscope available? | 0. No  1. Yes, seen functioning  2. Yes, reported functioning  3. Yes, not functioning | 1 or 2 = 1 point | Both reported and observed included |
|  | Do you have a thermometer available? | 0. No  1. Yes, seen functioning  2. Yes, reported functioning  3. Yes, not functioning | 1 or 2 = 1 point | Both reported and observed included |
|  | Do you have a scale available? | 0. No  1. Yes, seen functioning  2. Yes, reported functioning  3. Yes, not functioning | 1 or 2 = 1 point | Both reported and observed included |
|  | Do you have nasogastric tubes available? | 0. No  1. Yes, normally, but not today  2. Yes, observed, non expired  3. Yes, observed, expired  4. Yes, reported  5. Yes, observed, but no expiry date | 2 or 4 or 5 = 1 point | Both reported and observed included  Should have had same alternatives as scale above |
| Treatment | Do you have ORS sachets available? | 0. No  1. Yes, normally, but not today  2. Yes, observed, non expired  3. Yes, observed, expired  4. Yes, reported  5. Yes, observed, but no expiry date | 2 or 4 or 5 = 1 point | Both reported and observed included |
|  | Do you have a zinc tablets available? | 0. No  1. Yes, normally, but not today  2. Yes, observed, non expired  3. Yes, observed, expired  4. Yes, reported  5. Yes, observed, but no expiry date  97. I don’t know  99. No answer | 2 or 4 or 5 = 1 point | Both reported and observed included |
|  | Do you have Ringer's lactate or 0.9% NaCl (sodium chloride) available (intravenous treatment)? | 0. No  1. Yes, normally, but not today  2. Yes, observed, non expired  3. Yes, observed, expired  4. Yes, reported  5. Yes, observed, but no expiry date | 2 or 4 or 5 = 1 point | Both reported and observed included |
|  | Do you have Glucose 10% available? Or Do you have Glucose 50% available? | 0. No  1. Yes, normally, but not today  2. Yes, observed, non expired  3. Yes, observed, expired  4. Yes, reported  5. Yes, observed, but no expiry date | 2 or 4 or 5 = 1 point | Both reported and observed included |
|  | Do you have Ciprofloxacin available? | 0. No  1. Yes, normally, but not today  2. Yes, observed, non expired  3. Yes, observed, expired  4. Yes, reported  5. Yes, observed, but no expiry date | 2 or 4 or 5 = 1 point | Both reported and observed included |
| *’*Do not know’*, and ‘*no answer’* also possible answer as well as ‘*other*’ for some questions. | | | | |

**Supplement Table 3.** Knowledge questionnaire. Included categories and specific question for each category. Each correct answer gives one point. A score is generated for each category through dividing total point for category by number of questions for the categories x 100. Some questions use fraction scores (mentioned under coding) that in total add up to one point. All category scores are then summed together and divided by the total number of questions for a final mean score for each provider.

| Category | Question | Answers | Score coding | Comment |
| --- | --- | --- | --- | --- |
| General diarrhoea knowledge score | What is the definition of acute watery diarrhoea?? (Read alternatives out loud) | 1. Liquid stools two or more times/24 hours  2. Vomiting  3. Vomiting and loose stools  4. Liquid stools three or more times/24 hours  5. Liquid stools five or more times/24 hours | 4 = 1 point |  |
|  | What is the recommended treatment for diarrhoea (non bloody, non complicated, non cholera) in children? | 1. ORS  2. Home made ORS  3. Zinc  4. Antibiotic (e.g., metronidazol, ciprofloxacin...)  5. Antiemetic (to stop vomiting)  6. Paracetamol  7. Traditional medicine  8. Intravenous fluids  9. Vitamin B6  10.Vitamin A  11. Loprade (or other antidiarrheal)  12. Treatment for candida  13. Antiparasitic  14. Diadis | 1 & 3 = 1 point  1 & 3 + (anything else) = 0 |  |
|  | Can you tell me what ORS is? | 0. No  1. Yes – correct answer | 1 = 1 point | Correct definition:  A mixture of salts and glucose that must be mixed with drinking water to treat/prevent dehydration. |
|  | Can you show me how to prepare ORS? | 0. No  1. Yes – correct answer | 1 = 1 point | Correct definition:  Mix one packet of ORS with 1 litre of drinking water. (Some facilities may have a 500 ml packet, so it is possible to mix one packet with 500 ml of drinking water). |
| Dehydration classification score | A 3-year-old child has fever and diarrhoea. The diarrhoea started two days ago. The child is agitated. His eyes are not sunken. A skin pinch goes back slowly. He can drink. The child should be classified as: | 1. No dehydration  2. Light dehydration  3. Moderate dehydration  4. Severe dehydration | 3 = 1 point |  |
|  | To classify the dehydration status of a child with diarrhoea you will look at: (Read alternatives out loud) | 1. The general condition of the child (lethargic or unconscious, restless, and irritable)  2. For sunken eyes  3. Slowly resorbing skin pitch  4. For oedema of both feet  5. For palmar pallor  6. If the child is drinking eagerly or poorly  7. Dry mouth  8. Swollen abdomen  9. Cry without tears | 1,2,3,6,7,9 = 1/6 point  4,5,8= -1/3 point  Total of 1 point |  |
| Correct treatment score | Which of these medications did you prescribe to the last child you treated with diarrhoea? (Read alternatives out loud) | 1. ORS  2 Home made ORS  3. Zinc  4. Antibiotic (e.g., metronidazole,ciprofloxacin...)  5. Antiemetic (to stop vomiting)  6. Paracetamol  7. Traditional medicine  8. Intravenous fluids  9. Vitamin B6  10.Vitamin A  11. Loprade (or other antidiarrheoal)  12. Treatment for candida  13. Antiparasitic  14. Diadis | 1 & 3 = 1 point | There could be indication for other drugs depending on the case, thus inclusion of ORS and Zinc is sufficient. |
|  | When you are treating diarrhoea, which of these alternatives is your top priority? (Read alternatives out loud) | 1. Reduce the symptoms of diarrhoea  2. Decrease the duration of diarrhoea  3. Treat the cause  4. Avoiding dehydration | 4 = 1 point |  |
|  | When is antibiotic recommended in treatment of diarrhoea for children? (Read alternatives out loud) | 1. All types of diarrhoea  2. Diarrhoea with fever  3. Bloody diarrhoea  4. Diarrhoea without fever  5. Severe cholera | 3, 5 = 1/2 point  Total of 1 point |  |
|  | What is the recommended dosage of ORS for a 3 year old child with light dehydration? (Read alternatives out loud) | 1. After each episode of diarrhoea, 100-200 ml  2. When the child is thirsty, 100-200 ml  3. After each episode of diarrhoea, 10-20 ml.  4. After each fever episode 10-20 ml | 1 = 1 point |  |
|  | Should you give additional ORS? (child in previous question) (Read alternatives out loud) | 1. No, additional ORS can be dangerous  2. Yes, each time the child wants | 2 = 1 point |  |
|  | A child suffers from moderate dehydration. What is the preferred treatment? (Read alternatives out loud) | 1. ORS after each diarrhoea episode  2. ORS according to plan B  3. IV fluids according of plan B  4. IV fluids according of plan C  5. ORS on demand | 2 or 3 = 1 point |  |
|  | What do you do when the treatment is finished (child in previous question)? (Read alternatives out loud) | 1. Nothing  2. Give the same treatment again  3. Move from treatment plan B to plan A  4. Re-evaluate the child  5. Move from treatment plan B to plan C | 4 = 1 point |  |
|  | A 4-year-old comes to your clinic. She has not been able to drink or eat and for the last twenty for hours. Her caregiver has tried to give her ORS at home, but she has not succeeded, they have also tried again at the clinic after instructions from a nurse, but she still doesn’t succeed. The girl is TIRED but awake. She has dry lips but a normal skin-pinch. What is the preferred option?  (Read alternatives out loud) | 1. ORS orally  2. ORS via nasogastric tube  3. IV-fluids | 2 = 1 point |  |
|  | What is the daily need of fluids for a child with a weight of 12 kg with no extra losses. (Read alternatives out loud) | 1.200 ml  2.600 ml  3.1100 ml  4.1800 ml  5.2500 ml | 3 = 1 point |  |
|  | Which of the following lab tests is the most important in a child with diarrhoea that is very tired? (Read alternatives out loud) | 1. Stool sample  2. Sputum sample  3. Leukocytes  4. Glucose  5. Haemoglobin | 4 = 1 point |  |
| Advice score | For children that are breastfed: what are your recommendations regarding breastfeeding during diarrhoea? (Read alternatives out loud) | 1. No breastfeeding  2. Less breastfeeding  3. Normal breastfeeding  4. More breastfeeding | 3 or 4 = 1 point |  |
|  | What is your recommendation for food-intake in children with diarrhoea? The child does not vomit. (Read alternatives out loud) | 1. No food intake  2. Less food intake  3. Normal food intake  4. More food intake | 3 = 1 point |  |
|  | How should a child drink during diarrhoea? (Read alternatives out loud) | 1. Don’t drink at all  2. Drink less  3. Drink as normal  4. Drink more | 4 = 1 point |  |
|  | A child with diarrhoea is well enough to be treated at home. When do you recommend the caregiver to bring the child back to the facility? (Read alternatives out loud) | 1. The child is not able to breastfeed or drink  2. Becomes sicker  3. Develops a fever  4. Has blood in the stool | 1,2,3,4 = 1 subpoint  Total of 1 point |  |
| Intra venous fluid score | Select the three clinical signs of shock (according to WHO). (Read alternatives out loud) | 1. Cough  2. Capillary refill time > 3 sec.  3. Profuse diarrhoea  4. Fever  5. Weak and fast pulse  6. Runny nose  7. Cold extremities | 2,5,7= 1/3 point  1,3,4,6= - 1/4 point  Total of 1 point |  |
|  | What is the treatment for diarrhoea with severe dehydration for a child over 1 year old that is not in shock? (Read alternatives out loud) | 1.30 ml i.v. during one hour followed by 100 ml during 7 hours  2.20 ml i.v. during 30 minutes followed by 30 ml pendant 2.5 hours.  3.30 ml/kg during 30 minutes followed by 70 ml/kg pendant 2.5 hours  4.30 ml/kg during one hour followed by 20 ml/kg during 2.5 hours. | 3 = 1 Point |  |
|  | In a child with shock and NO MALNUTRITION a child should be given the following treatment fluid: (Read alternatives out loud) | 1. 10-20 ml/kg IV infusion over 30-60 min  2. 10-20 ml/kg IV infusion over 90-120 min  3. 30-40 ml/kg IV infusion over 30-60 min  4. Maintenance fluid only | 1 = 1 point |  |
|  | Should fluids with or without electrolytes be given for shock treatment and rehydration? (Read alternatives out loud) | 0. With  1. Without | 1 = 1 point |  |
| Prevention score | Which are risk factors for diarrhoea?  & How can we prevent that children fall ill from diarrhoea? (Do not read out loud) | 1. Ingestion of contaminated water  2. Ingestion of contaminated food  3. Contact with people who have died of the disease  4. Lack of sanitary systems  5. Contact with body fluids from people with the disease  6. Living in the same household as a case  7. Bad hand hygiene  8. Not exclusively breastfed during first 6 months of life  9. Wash hands with soap and water  10. Cook food thoroughly  11. Wash vegetables/fruits before eating  12. Dispose of human waste properly  13. Boil water or treat water with chlorine before drinking  14. Clean cooking utensils/vessels  15. Cholera vaccine  16. Rota virus vaccine  17. Cannot prevent | 1,2,3,4,5,6,7,8,  9,10,11,12,13,14,  15,16,17=0,25 sub points  Max 2 points | Here 2 points is possible since this is a combination of two separate questions. |
|  | At which of these occasions should you clean your hands? (Do not read out loud) | 1. Before touching a patient  2. Before clean/antiseptic procedure  3. After body fluid exposure risk  4. After touching a patient  5. After touching patient surroundings | 1,2,3,4,5=0,2 sub points  Max 1 point |  |
| Cholera score | Cholera vaccines offer complete protection, with effectiveness increasing with time? (Read alternatives out loud) | 1. True, protection is permanent  2. False, protection is time-dependent | 2 = 1 point |  |
|  | What is the definition of a suspected cholera case during an ongoing outbreak? (Read alternatives out loud) | 1. Any person over 2 years of age who develops severe dehydration or dies from acute watery diarrhoea  2. Any person with acute watery diarrhoea  3. Any person over 1 year of age with cough, fever and diarrhoea  4. Any person over 1 year of age with acute watery diarrhoea | 2 or 4= 1 point | 4 is National Cholera Program definition. |
|  | In which of these patients should you suspect cholera  if no outbreak is ongoing? (Read alternatives out loud) | 1. A patient over the age of 2 who develops severe dehydration or dies from acute watery diarrhoea  2. A 3 year old patient with moderate dehydration  3. A 1-year-old child with cough, fever and diarrhoea.  4. Anyone with acute watery diarrhoea | 1 = 1 point |  |
| *’Do not know’, and ‘no answer’ also possible answer as well as other for some questions. | | | | |

**Group discussion guide**

**(doctors/nurses/drug shop vendors/traditional health practitioners)***

1. *Welcome to the participants*
2. *Informed consent*
   - Ensure that everyone is aware of the audio recording
   - Assign random numbers to participants and reiterate confidentiality
   - Filling in the participant information sheet
3. *Introductory question (don't stay too long on this question)*
   - Can you tell me what kind of sick children you normally see in your environment?
     - Probe: How many children do you see? How many have diarrhoea?
4. *Paediatric diarrhoea*
   - What do you know about diarrhoea in children?
     - Probes:
       1. Cause
       2. Presentation
       3. Main groups affected

*(INFORMATION FOR THE INTERVIEWER: MAKE SURE THAT ALL PARTICIPANTS UNDERSTAND WHAT WE MEAN BY DIARRHOEA: LOOSE STOOLS (WITH OR WITHOUT VOMITING))*

- - Can you describe a typical case of diarrhoea in your setting?
  - How do you normally diagnose a child with diarrhoea?
    - Probes:
      1. Specific questions asked?
      2. Specific clinical signs sought?
      3. Tools used (guidelines)?
  - How do you decide if it is a severe or non-severe case of diarrhoea?
    - Probe:
      1. Does malnutrition change the assessment?
  - How do you assess whether the child is dehydrated?
    - Probes:
      1. Specific questions asked?
      2. Specific clinical signs sought?
      3. Tools used (guidelines)?
  - Can you describe how you treat a child with diarrhoea?
    - Probes:
      1. Drug used and when
      2. Fluids administered - when, how and what type
      3. When to use intravenous fluids
      4. Things not to give to children (e.g. medication, types of food etc.)
      5. Does malnutrition change the way you manage?
  - Are there times when you deviate from what you consider to be proper care? *(Here we want to know about limiting factors (e.g. lack of medication, parents cannot pay). We are not looking for reasons for incorrect treatment)*
    - Probes:
      1. In what way?
      2. What are the reasons for this?
  - How do economic incentives influence the type of medicines you recommend for treating diarrhoea in children?
    - If you get free treatment from an NGO for example. Would this make you more or less likely to prescribe the drug?

1. *Economic incentives and access to medicines* [DRUG SHOP VENDORS ONLY]
   - How do you decide what type of medicine you have in stock to treat diarrhoea in children?
   - What would increase your tendency to prescribe Oral Rehydration Serum?
2. *Policy recommendations*
   - What are the main drivers and barriers in your efforts to address diarrhoea in children?
     - Probes :
       1. Catalysts (what works well)
       2. Obstacles (what doesn't work well)
   - What could be done in your facility to improve the management of diarrhoea in children?
   - What could be done by the community to improve the management of diarrhoea in children?
   - What could be done by the government to improve the management of diarrhoea in children?
   - What could be done by the external actors such as NGOs to improve the management of diarrhoea in children?
   - If you could choose one of these interventions (referring to the ones suggested), which would you prefer? Why or why not?
3. *Response to cholera*
   - Are you aware of any cholera interventions in the area where you work?
     - If so, can you describe them? When did they take place? By whom?
       1. How do you think these interventions were organised?
       2. Are there things you wish had been done differently?
     - If not, why do you think there are no initiatives in your area?
4. *Reflecting on one's own role in the protection, prevention and treatment of diarrhoea and in the fight against cholera.*
   - How do you see your role in protecting children from diarrhoea?
     - Probe:
       - Preventing children from getting sick
       - Home/institutional treatment
       - Knowledge of caregivers
   - How do you see your role in a cholera outbreak?
     - Do you think your role could be more effective during a cholera outbreak, and if so how?
5. *Perception of formal health care [ONLY TRADITIONAL HEALTH PRACTITIONERS]*
   - How do you see your role in treating children with diarrhoeal diseases in relation to the wider health care system (health centres, hospitals, etc.)?
   - How would you feel about working with the wider healthcare system to treat children with diarrhoeal diseases?
     - How would such collaboration ideally be implemented?
6. *Additional aspects*
   - Is there anything else about diarrhoea in children and what we have discussed today that we have not mentioned that you would like to add?

**Concluding question**

- Of all the things we have discussed today, what do you think are the most important issues?

**Conclusion**

- Thank you for your participation. This discussion has been very enriching.
- Your views will be a valuable asset to the study.
- We hope you found the discussion interesting.
- If you are not satisfied with something or wish to complain, please contact the local PI or speak to me later.

I would like to remind you that all of you will be anonymous in the report.

*** *Only qualitative data matching the quantitative categories, created in accordance with existing literature relevant to diarrhoeal disease management and cholera response was included in the study. A separate qualitative study exploring the remaining data will be published elsewhere.*

*References*

1. Sustaining Health Outcomes through the Private Sector Plus Project. Democratic Republic of the Congo Private Health Sector Assessment. Brief. Rockville, MD, 2019.

2. World Health Organization. Service Availability and Readiness Assessment (SARA): An Annual Monitoring System for Service Delivery. Geneva, 2015.


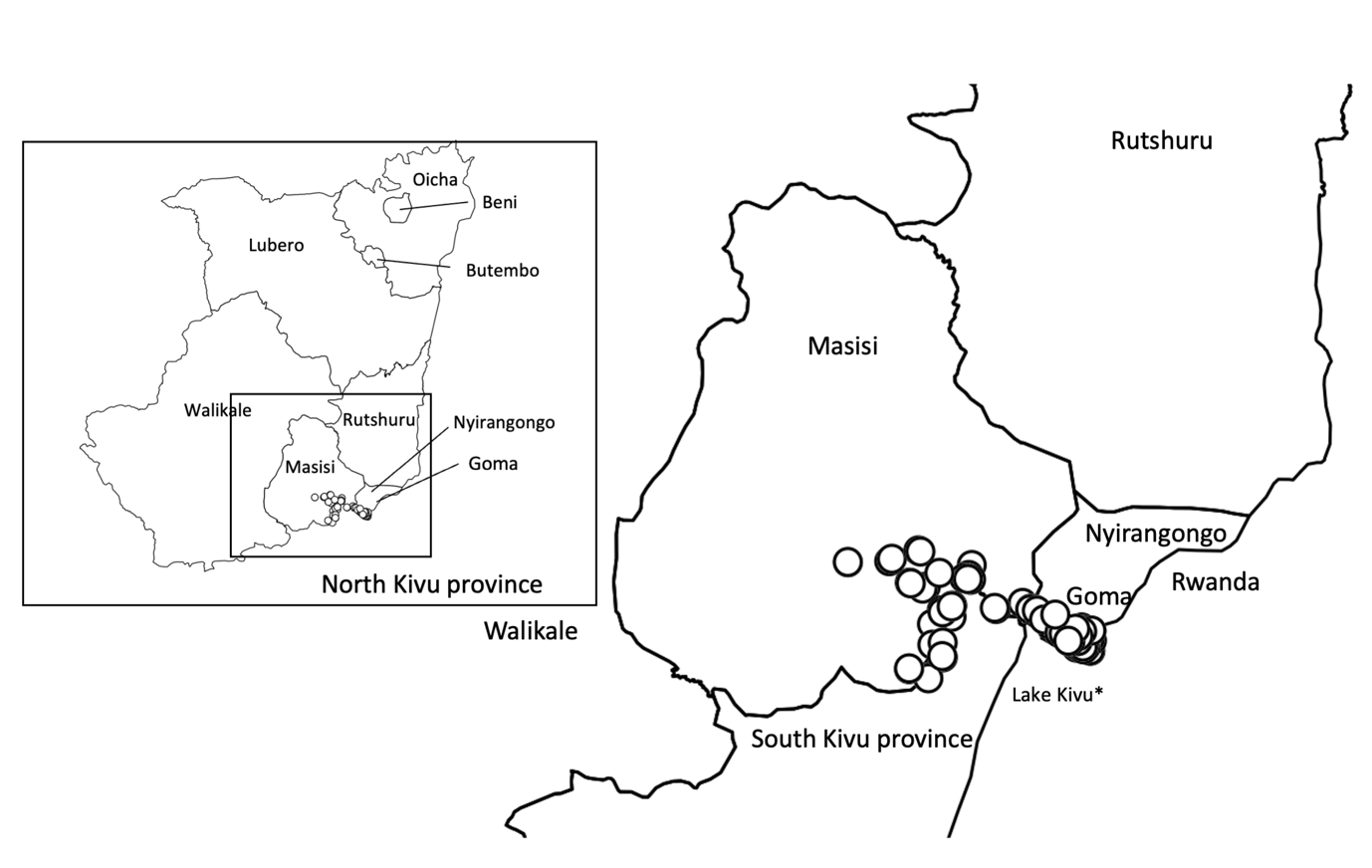


**Supplement Figure 1.** Map with included facilities for the North Kivu province plotted. *Lake Kivu is not included on the map, and neither is country borders.


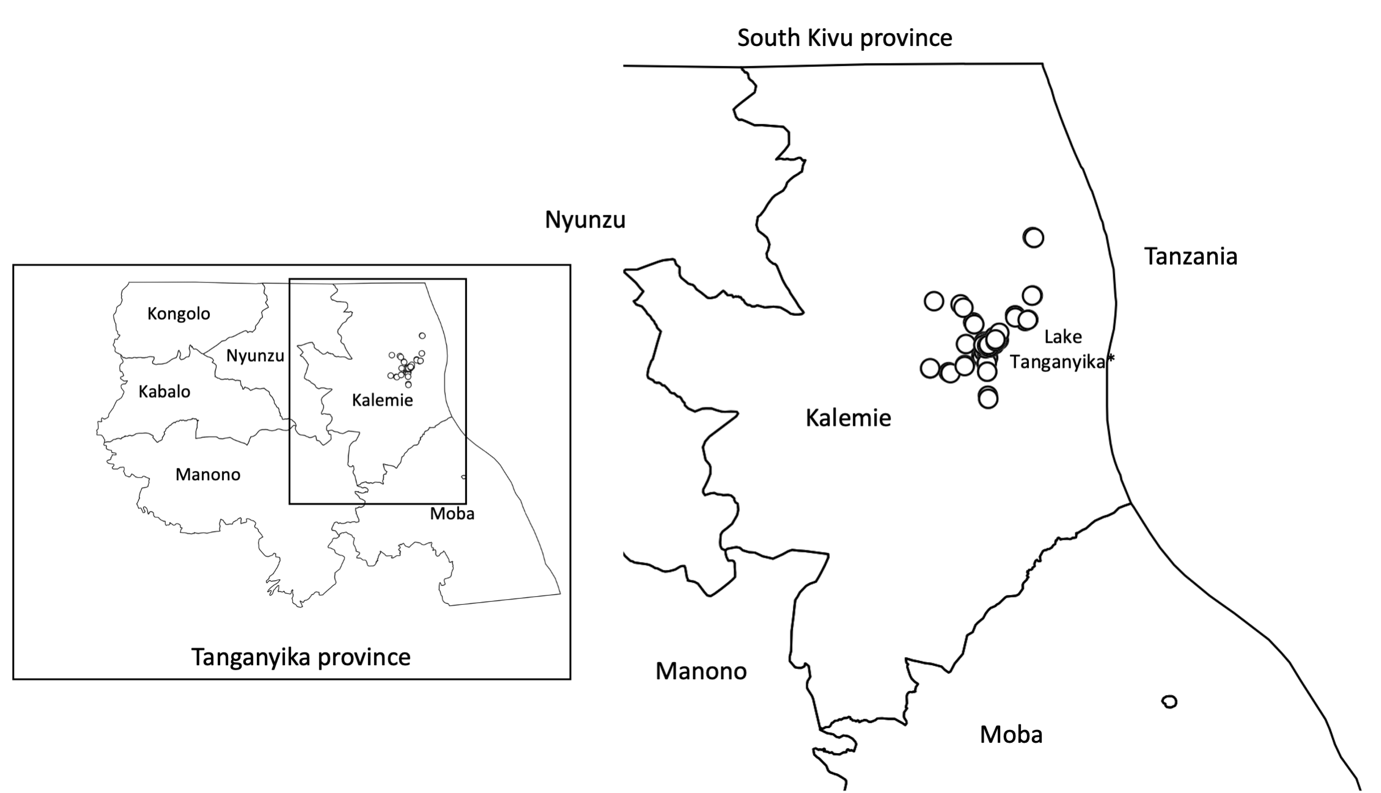


**Supplement Figure 2.** Map with included facilities for the Tanganyika province plotted. *Lake Tanganyika is not included on the map, and neither is country borders.
