# Supplementary material for: Facility capacity and provider knowledge for cholera surveillance and diarrhoea case management in cholera hotspots in the Democratic Republic of Congo – a mixed-methods study
Source: Glob Health Action. 2024 Mar 5;17(1):2317774. doi: 10.1080/16549716.2024.2317774 (PMC10916892; doi:10.1080/16549716.2024.2317774)
Supplement: Supplementary material 2 DRC Diarrhoea GHA_.docx [file ZGHA_A_2317774_SM2507.docx]

**Supplementary material 2**

**Supplement Table 4.** Summary of included facilities for audit questionnaire. Extend version of Table 1a. in manuscript.

|  | Health facilities  (N =165) | | | | | | | | |  |
| --- | --- | --- | --- | --- | --- | --- | --- | --- | --- | --- |
|  | Hospital  (n = 82) | | | Health centre  (n = 52) | | | Health post  (n = 31) | | | Drug  shop  (N = 79) |
| Ownership | Public | Private | Confessional | Public | Private | Confessional | Public | Private | Confessional |  |
| Total (%) | 26 (16) | 50 (30) | 6 (4) | 44 (27) | 4 (2) | 4 (2) | 6 (4) | 24 (15) | 1 (1) | 79 (100) |
| Urban (%) | 22 (17) | 49 (38) | 6 (5) | 20 (15) | 4 (3) | 4 (3) | 4 (3) | 20 (15) | 1 (1) | 44 (56) |
| Rural (%) | 4 (11) | 1 (3) | 0 | 24 (69) | 0 (0) | 0 (0) | 2 (6) | 4 (11) | 0 (0) | 35 (44) |
| Median number of persons working in facility (IQR) | 24 (11,40) | 9 (7,13) | 28 (24,45) | 10 (8,15) | 3 (2,3) | 8 (6,11) | 6 (4,7) | 3 (2,4) | 5 (N/A) |  |
| Median number of consultations per year (IQR) | 2264 (500, 5488) | 793 (338. 1860) | 3840 (2264,4216) | 5401 (3360,  8219) | 360 (240,1600) | 406 (256,6746) | 1800  (720, 2253) | 372 (300,800) | 168 (N/A) |  |
| Median number of <5 years ambulatory consultations per day (excluding, vaccinations, growth monitoring) (IQR) | 5 (4,17) | 3 (2,4) | 2 (N/A) | 6 (5,14) | 18 (3,35) | 103 (21,305) | 7 (3,15) | 3 (1,8) | 7 (3,10) |  |
| Median number of diarrhoea cases in last 30 days (IQR) | 16 (6,36) | 10 (5,19) | 13 (5,19) | 28 (10,49) | 33 (12,35) | 20 (10,33) | 14 (5,35) | 4 (7,20) | 0 (N/A) |  |
| Currently receives any type of support | 37% | 1% | 80% | 55% | 0% | 75% | 17% | 4% | 0% |  |
| Free or flat rate price for diarrhoea | 46% | 8% | 17% | 68% | 0% | 50% | 50% | 13% | 100% |  |

**Supplement Table 5.** Linear regression analysis of total audit score.

| Variable | Crude coefficient  (95% CI) | Adjusted coefficient*  (95% CI) |
| --- | --- | --- |
| Facility type |  |  |
| Hospital | *Ref* | *Ref* |
| Health centre | 7.8 (2.7; 12.9) | 0.2 (-4.8; 5.3) |
| Health post | -18.4 (-24.4; -12.3) | -13.0 (-18.1; -7.8) |
| Setting |  |  |
| Urban | *Ref* | *Ref* |
| Rural | 6.1 (-0.3; 12.5) | -2.3 (-7.6; 3.0) |
| Ownership |  |  |
| Public | *Ref* | *Ref* |
| Private | -19.4 (-23.8; -14.9) | -11.6 (-16.7; -6.6) |
| Confessional | 4.7 (-4.1; 13.6) | 7.0 (-0.9; 14.8) |
| Any type of external support |  |  |
| No | *Ref* | *Ref* |
| Yes | 13.7 (8.9; 18.6) | 5.8 (1.8; 9.8) |
| Availability of cholera treatment centre |  |  |
| No | *Ref* | *Ref* |
| Yes | 20.2 (11.9; 28.6) | 13.3 (6.5; 20.0) |
| **Adjusted for facility type, setting, ownership, any type of external support in the previous three years, availability of cholera treatment centre, and province.* | | |

**Supplement Table 6.** Linear regression analysis of total knowledge score for health facility providers.

| Variable | Crude coefficient  (95% CI) | Adjusted coefficient*  (95% CI) |
| --- | --- | --- |
| Facility type |  |  |
| Hospital | *Ref* | *Ref* |
| Health centre | 1.3 (-2.2; 4.8) | 0.9 (-3.4; 5.1) |
| Health post | -1.7 (-5.8; 2.5) | -0.7 (-5.0; 3.6) |
| Profession |  |  |
| Medical doctor | *Ref* | *Ref* |
| Nurse | -5.7 (-10.8; -0.7) | -5.3 (-10.6; 0.1) |
| Other | -10.3 (-18.5; -2.1) | -11.7 (-20.0; -3.3) |
| Age |  |  |
| 18-30 | *Ref* | *Ref* |
| 31-50 | 4.5 (1.2; 7.8) | 4.1. (0.7; 7.6) |
| >50 | 4.8 (-0.3; 9.8) | 4.1 (-1.0; 9.3) |
| Ownership |  |  |
| Public | *Ref* | *Ref* |
| Private | -3.3 (-6.4; -0.2) | -2.8 (-6.8; 1.2) |
| Confessional | 2.8 (-3.6; 9.2) | 1.8 (-4.7; 8.3) |
| Setting |  |  |
| Urban | Ref | Ref |
| Rural | 0.9 (-2.8; 4.6) | 0.1 (-4.0; 4.3) |
| Received training previous three years |  |  |
| No | *Ref* | Ref |
| Yes | 2.7 (-0.8; 6.1) | 2.3 (-1.3; 6.0) |
| **Adjusted for facility type, profession, age, ownership, setting, training in previous three years, and province.* | | |

**Audit sub scores**

**WASH**

**Supplement Table 7.** Logistic regression analysis of WASH audit score.

| Variable | Crude coefficient  (95% CI) | Adjusted coefficient*  (95% CI) |
| --- | --- | --- |
| Facility type |  |  |
| Hospital | Ref | Ref |
| Health centre | 0.8 (0.4; 1.6) | 0.4 (0.1; 1.4) |
| Health post | 0.2 (0.1; 0.4) | 0.3 (0.1; 0.8) |
| Setting |  |  |
| Urban | Ref | Ref |
| Rural | 0.5 (0.3; 1.2) | 0.2 (0.1; 0.7) |
| Ownership |  |  |
| Public | Ref | Ref |
| Private | 0.3 (0.1; 0.5) | 0.1 (0.0; 0.4) |
| Confessional | 1.0 (0.3; 4.2) | 0.4 (0.1; 2.4) |
| Any type of external support |  |  |
| No | Ref | Ref |
| Yes | 2.8 (1.5; 5.3) | 1.7 (0.8; 3.6) |
| Availability of cholera treatment centre |  |  |
| No | Ref | Ref |
| Yes | 0.9 (0.3; 2.7) | 0.7 (0.2; 2.8) |
| *Adjusted for facility type, setting, ownership, any type of external support in the previous three years, availability of cholera treatment centre, and province. | | |

**IPC score**

**Supplement Table 8.** Logistic regression analysis of IPC audit score.

| Variable | Crude coefficient  (95% CI) | Adjusted coefficient*  (95% CI) |
| --- | --- | --- |
| Facility type |  |  |
| Hospital | Ref | Ref |
| Health centre | 0.9 (0.4; 2.1) | 0.9 (0.3; 3.2) |
| Health post | 0.1 (0.1; 0.9) | 0.2 (0.0; 2.1) |
| Setting |  |  |
| Urban | Ref | Ref |
| Rural | 0.5 (0.2; 1.5) | 0.1 (0.0; 0.8) |
| Ownership |  |  |
| Public | Ref | Ref |
| Private | 0.4 (0.2; 0.9) | 0.6 (0.2; 2.3) |
| Confessional | 2.7 (0.7; 9.9) | 3.1 (0.6; 16.0) |
| Any type of external support |  |  |
| No | Ref | Ref |
| Yes | 4.2 (1.6; 10.8) | 2.5 (0.8; 7.3) |
| Availability of cholera treatment centre |  |  |
| No | Ref | Ref |
| Yes | 4.1 (1.4; 11.9) | 14.8 (2.2; 97.9) |
| *Adjusted for facility type, setting, ownership, any type of external support in the previous three years, availability of cholera treatment centre, and province. | | |

**Cholera surveillance score**

**Supplement Table 9.** Logistic regression analysis of cholera surveillance score.

| Variable | Crude coefficient  (95% CI) | Adjusted coefficient*  (95% CI) |
| --- | --- | --- |
| Facility type |  |  |
| Hospital | Ref | Ref |
| Health centre | 2.1 (0.5; 8.1) | 0.2 (0.0; 2.5) |
| Health post | - | - |
| Setting |  |  |
| Urban | Ref | Ref |
| Rural | 8.8 (2.1; 37.1) | 1.7 (0.1; 22.4) |
| Ownership |  |  |
| Public | Ref | Ref |
| Private | - | - |
| Confessional | - | - |
| Any type of external support |  |  |
| No | Ref | Ref |
| Yes | - | - |
| Availability of cholera treatment centre |  |  |
| No | Ref | Ref |
| Yes | 148.0 (16.4; 1332.0) | 99.4 (4.3; 2288.0) |
| **Based on 52/165 observations due to collinearity. No private (0/78) or confessional (0/11) facilities met the 70% threshold for the cholera surveillance score compared to 9/76 for public facilities. None of the facilities without external support met the same criteria (0/73) compared to 9/92 of the facilities that had received support. Also adjusted for province due to different data collection teams.* | | |

**Guideline score**

**Supplement Table 10.** Logistic regression analysis of guideline score.

| Variable | Crude coefficient  (95% CI) | Adjusted coefficient*  (95% CI) |
| --- | --- | --- |
| Facility type |  |  |
| Hospital | Ref | Ref |
| Health centre | 2.2 (1.0; 4.9) | 0.8 (0.3; 2.5) |
| Health post | - | - |
| Setting |  |  |
| Urban | Ref | Ref |
| Rural | 3.3 (1.4; 7.7) | 0.8 (0.2; 3.1) |
| Ownership |  |  |
| Public | Ref | Ref |
| Private | 0.1 (0.0; 0.3) | 0.1 (0.0; 0.5) |
| Confessional | 0.3 (0.0; 1.7) | 0.3 (0.0; 2.7) |
| Any type of external support |  |  |
| No | Ref | Ref |
| Yes | 1.5 (0.7; 3.3) | 0.3 (0.1; 1.1) |
| Availability of cholera treatment centre |  |  |
| No | Ref | Ref |
| Yes | 21.8 (6.4; 75.0) | 17.7 (3.6; 88.0) |
| *Adjusted for facility type, setting, ownership, any type of external support in the previous three years, availability of cholera treatment centre, and province. | | |

**Community engagement score**

**Supplement Table 11.** Logistic regression analysis of community engagement score.

| Variable | Crude coefficient  (95% CI) | Adjusted coefficient*  (95% CI) |
| --- | --- | --- |
| Facility type |  |  |
| Hospital | Ref | Ref |
| Health centre | 3.8 (1.6; 9.1) | 1.3 (0.3; 5.6) |
| Health post | 1.1 (0.3; 3.7) | 1.4 (0.5; 4.0) |
| Setting |  |  |
| Urban | Ref | Ref |
| Rural | 5.0 (2.2; 11.6) | 2.5 (0.9; 7.5) |
| Ownership |  |  |
| Public | Ref | Ref |
| Private | 0.1 (0.0; 0.4) | 0.2 (0.1; 0.8) |
| Confessional | 1.2 (0.3; 4.6) | 2.9 (0.7; 12.8) |
| Any type of external support |  |  |
| No | Ref | Ref |
| Yes | 2.0 (0.9; 4.5) | 1.0 (0.4; 2.8) |
| Availability of cholera treatment centre |  |  |
| No | Ref | Ref |
| Yes | 9.6 (3.2; 29.2) | 4.6 (1.3; 16.3) |
| *Adjusted for facility type, setting, ownership, any type of external support in the previous three years, availability of cholera treatment centre, and province. | | |

**Equipment score**

**Supplement Table 12.** Logistic regression analysis of equipment score.

| Variable | Crude coefficient  (95% CI) | Adjusted coefficient*  (95% CI) |
| --- | --- | --- |
| Facility type |  |  |
| Hospital | Ref | Ref |
| Health centre | 0.2 (0.1; 0.5) | 0.1 (0.0; 0.3) |
| Health post | 0.4 (0.0; 0.3) | 0.0 (0.0; 0.4) |
| Setting |  |  |
| Urban | Ref | Ref |
| Rural | 0.4 (0.1; 1.0) | 0.5 (0.1; 2.0) |
| Ownership |  |  |
| Public | Ref | Ref |
| Private | 0.6 (0.3; 1.2) | 0.4 (0.1; 1.0) |
| Confessional | 6.1 (1.5; 25.3) | 10.1 (1.5; 68.2) |
| Any type of external support |  |  |
| No | Ref | Ref |
| Yes | 3.6 (1.7; 7.8) | 3.0 (1.2; 7.6) |
| Availability of cholera treatment centre |  |  |
| No | Ref | Ref |
| Yes | 1.6 (0.5; 4.6) | 2.5 (0.5; 12.1) |
| *Adjusted for facility type, setting, ownership, any type of external support in the previous three years, availability of cholera treatment centre, and province. | | |

**Treatment score**

**Supplement Table 13.** Logistic regression analysis of treatment score.

| Variable | Crude coefficient  (95% CI) | Adjusted coefficient  (95% CI) |
| --- | --- | --- |
| Facility type |  |  |
| Hospital | Ref | Ref |
| Health centre | 0.9 (0.5; 1.9) | 1.1 (0.4; 3.0) |
| Health post | 0.3 (0.1; 0.7) | 0.4 (0.2; 1.2) |
| Setting |  |  |
| Urban | Ref | Ref |
| Rural | 0.6 (0.3; 1.2) | 0.4 (0.2; 1.3) |
| Ownership |  |  |
| Public | Ref | Ref |
| Private | 0.7 (0.3; 1.2) | 0.9 (0.3; 2.4) |
| Confessional | 2.9 (0.6; 14.5) | 2.2 (0.4; 13.1) |
| Any type of external support |  |  |
| No | Ref | Ref |
| Yes | 1.9 (1.0; 3.6) | 1.8 (0.8; 3.9) |
| Availability of cholera treatment centre |  |  |
| No | Ref | Ref |
| Yes | 0.6 (0.2; 1.6) | 0.6 (0.2; 2.1) |
| *Adjusted for facility type, setting, ownership, any type of external support in the previous three years, availability of cholera treatment centre, and province. | | |

**Knowledge score regressions including all provider groups**

**Total knowledge**

**Supplement Table 14.** Linear regression analysis including all provider groups of total knowledge score.

| Variable | Crude coefficient  (95% CI) | Adjusted coefficient*  (95% CI) |
| --- | --- | --- |
| Profession |  |  |
| Medical doctor | Ref | Ref |
| Nurse | -5.7 (-11.3; -0.2) | -4.3 (-9.9; 1.2) |
| Other health facility staff | -10.3 (-19.4; -1.2) | -9.7 (-18.7; -0.7) |
| Drug shop vendor | -18.7 (-24.5; -12.9) | -16.6 (-22.5; -10.8) |
| Traditional health practitioner | -27.4 (-33.2; -21.6) | -26.0 (-31.9; -20.0) |
| Age |  |  |
| 18-30 | Ref |  |
| 31-50 | 2.3 (-1.3; 5.9) | 4.1 (1.4; 6.9) |
| >50 | -4.6 (-9.4; 0.2) | 2.7 (-1.2; 6.6) |
| Setting |  |  |
| Urban | Ref | Ref |
| Rural | -6.1 (-9.4; -2.8) | -0.7 (-3.3; 1.9) |
| Received training previous three years |  |  |
| No | Ref | Ref |
| Yes | 6.6 (2.7; 10.6) | 3.7 (0.6; 6.8) |
| **Adjusted for profession, age, setting, and training in the previous three years, and province.* | | |

**Price**

**Supplement Table 15.** Price of drugs.

| Variable | Observations  (n) | Mean  (CDF*) | Std. Dev.  (CDF*) | Min  (CDF*) |
| --- | --- | --- | --- | --- |
| Price 1L ORS | 142 | 490 | 285 | 104 |
| Price 10 Zinc tablets 20 mg | 111 | 906 | 415 | 300 |
| Profit 1L ORS | 141 | 480 | 751 | 20 |
| Profit 10 Zinc tablets 20 mg | 114 | 562 | 590 | 100 |
| Profit 10 Ciprofloxacin tablets 500 mg | 141 | 757 | 799 | 100 |
| Profit 10 Metronidazole tablets 500 mg | 146 | 532 | 420 | 50 |
| Profit 10 tablets Loperamide 2 mg | 93 | 356 | 401 | 50 |
| *CDF *abbreviation for local currency Congolese Franc* | | | | |

**Missing data imputation**

**Supplement Table 16.** Methods used and number of missing values imputed for variables with missing data.

| **Variable**  *Strata* | Total number of observations for strata | Number imputed | Max possible difference from true proportion due to imputation (%) | Imputation method used |
| --- | --- | --- | --- | --- |
| Audit | | | | |
| **Separate toilet for patients** |  |  |  |  |
| *Public health post* | 6 | 1 | 17 | Imputed as 0 |
| *Private health post* | 24 | 1 | 4 | Imputed as 0 |
| **Safe disposal of infectious body fluids** |  |  |  |  |
| *Public health post* | 6 | 1 | 17 | Imputed as 0 |
| *Private health post* | 24 | 1 | 4 | Imputed as 0 |
| *Public hospital* | 26 | 1 | 4 | Imputed as 0 |
| **Safe disposal of soft waste** |  |  |  |  |
| *Private health post* | 24 | 2 | 8 | Imputed as 0 |
| **Water and soap nearby toilet** |  |  |  |  |
| *Public health post* | 6 | 1 | 17 | Imputed as 0 |
| *Private health post* | 24 | 1 | 4 | Imputed as 0 |
| **Water and soap clinical care setting** |  |  |  |  |
| *Drug shops* | 79 | 11 | 14 | Imputed as 0 |
| **Chlorine for cleaning purposes** |  |  |  |  |
| *Drug shops* | 79 | 1 | 1 | Imputed as 0 |
| **Availability of cholera notification form** |  |  |  |  |
| *Drug shops* | 79 | 2 | 3 | Imputed as 0 |
| **Availability of internet or phone to transfer health data** |  |  |  |  |
| *Drug shops* | 79 | 1 | 1 | Imputed as 0 |
| **Cholera definition on wall** |  |  |  |  |
| *Drug shops* | 79 | 1 | 1 | Imputed as 0 |
| **Availability of nasogastric tube** |  |  |  |  |
| *Private health post* | 24 | 3 | 13 | Imputed mode stratified by facility type and ownership |
| *Public health centre* | 44 | 6 | 14 | Imputed mode stratified by facility type and ownership |
| *Public hospital* | 26 | 1 | 4 | Imputed mode stratified by facility type and ownership |
| *Private hospital* | 50 | 1 | 2 | Imputed mode stratified by facility type and ownership |
| *Confessional hospital* | 6 | 2 | 30 | Imputed mode stratified by facility type and ownership |
| *Drug shops* | 79 | 3 | 4 | Imputed mode stratified by facility type and ownership |
| Knowledge questionnaire | | | | |
| **Definition acute watery diarrhoea** |  |  |  |  |
| *Drugstore vendor* | 75 | 1 | 1 | Imputed as 0 |
| **Can explain what ORS is** |  |  |  |  |
| *Traditional health practitioner* | 73 | 1 | 1 | Imputed as 0 |
| **Can explain how to prepare ORS** |  |  |  |  |
| *Traditional health practitioner* | 73 | 1 | 1 | Imputed as 0 |
| **Can identify moderate dehydration** |  |  |  |  |
| *Drugstore vendor* | 75 | 3 | 4 | Imputed as 0 |
| *Traditional health practitioner* | 73 | 1 | 1 | Imputed as 0 |
| **Prescribed ORS and zinc to last child with diarrhoea** |  |  |  |  |
| *Drug shop vendor* | 73 | 2 | 3 | Imputed as 0 |
| **Knows main priority with diarrhoea treatment** |  |  |  |  |
| *Drugstore vendor* | 75 | 1 | 1 | Imputed as 0 |
| **Knows indication for antibiotics** |  |  |  |  |
| *Nurse* | 136 | 2 | 1 | Imputed as 0 |
| *Drugstore vendor* | 75 | 3 | 4 | Imputed as 0 |
| *Traditional health practitioner* | 73 | 4 | 5 | Imputed as 0 |
| **Knows treatment plan A** |  |  |  |  |
| *Nurse* | 136 | 4 | 3 | Imputed as 0 |
| *Drugstore vendor* | 75 | 2 | 3 | Imputed as 0 |
| *Traditional health practitioner* | 73 | 3 | 4 | Imputed as 0 |
| **Knows indication for additional ORS** |  |  |  |  |
| *Drugstore vendor* | 75 | 1 | 1 | Imputed as 0 |
| *Traditional health practitioner* | 73 | 1 | 1 | Imputed as 0 |
| **Knows stage of dehydration to use Plan B** |  |  |  |  |
| *Drugstore vendor* | 75 | 1 | 1 | Imputed as 0 |
| **Is aware of the importance to re-evaluate the child** |  |  |  |  |
| *Drugstore vendor* | 75 | 1 | 1 | Imputed as 0 |
| **Can identify when nasogastric tube is preferred** |  |  |  |  |
| *Nurse* | 136 | 1 | 1 | Imputed as 0 |
| *Drugstore vendor* | 75 | 4 | 5 | Imputed as 0 |
| *Traditional health practitioner* | 73 | 7 | 10 | Imputed as 0 |
| **Knows the basal fluid need for a 3-year-old 12 kg child** |  |  |  |  |
| *Medical doctor* | 16 | 1 | 6 | Imputed as 0 |
| *Nurse* | 136 | 8 | 6 | Imputed as 0 |
| *Traditional health practitioner* | 73 | 3 | 4 | Imputed as 0 |
| **Knows indication to verify glucose level** |  |  |  |  |
| *Drugstore vendor* | 75 | 8 | 11 | Imputed as 0 |
| *Traditional health practitioner* | 73 | 5 | 7 | Imputed as 0 |
| **Correct advice regarding breastfeeding** |  |  |  |  |
| *Nurse* | 136 | 1 | 1 | Imputed as 0 |
| *Drugstore vendor* | 75 | 1 | 1 | Imputed as 0 |
| *Traditional health practitioner* | 73 | 2 | 3 | Imputed as 0 |
| **Correct advice regarding food-intake** |  |  |  |  |
| *Traditional health practitioner* | 73 | 1 | 1 | Imputed as 0 |
| **Correct advice when to return to facility** |  |  |  |  |
| *Nurse* | 136 | 3 | 2 | Imputed as 0 |
| *Drugstore vendor* | 75 | 2 | 3 | Imputed as 0 |
| *Traditional health practitioner* | 73 | 2 | 3 | Imputed as 0 |
| **Knows WHO clinical definition of choc** |  |  |  |  |
| *Nurse* | 136 | 1 | 1 | Imputed as 0 |
| *Drugstore vendor* | 73 | 1 | 1 | Imputed as 0 |
| **Knows treatment plan C** |  |  |  |  |
| *Nurse* | 127 | 7 | 6 | Imputed as 0 |
| **Knows treatment of choc** |  |  |  |  |
| *Nurse* | 127 | 4 | 3 | Imputed as 0 |
| **Know that IV-fluids should contain electrolytes** |  |  |  |  |
| *Nurse* | 127 | 2 | 2 | Imputed as 0 |
| **Knows that vaccinations effect reduces with time** |  |  |  |  |
| *Nurse* | 136 | 1 | 1 | Imputed as 0 |
| *Drugstore vendor* | 75 | 1 | 1 | Imputed as 0 |
| **Knows definition of cholera when no outbreak is ongoing** |  |  |  |  |
| *Nurse* | 136 | 9 | 7 | Imputed as 0 |
| *Drugstore vendor* | 75 | 7 | 9 | Imputed as 0 |
| **Knows definition of cholera case during an outbreak** |  |  |  |  |
| *Nurse* | 136 | 4 | 3 | Imputed as 0 |
| *Drugstore vendor* | 75 | 8 | 11 | Imputed as 0 |
| *Traditional health practitioner* | 73 | 1 | 1 | Imputed as 0 |

**Sensitivity analysis**

*Excluding variables from regression with more missing data than 15% (separate toilets for patients; safe disposal of infectious body fluids water and soap nearby toilet)*

***Total audit score***

**Supplement Table 17.** Linear regression analysis of WASH audit score.

| Variable | Adjusted coefficient*  (95% CI) |
| --- | --- |
| Facility type |  |
| Hospital | Ref |
| Health centre | -12.3 (-17.3; -7.2) |
| Health post | 0.1 (-4.8; 5.1) |
| Setting |  |
| Urban | Ref |
| Rural | -2.2 (-7.4; 3.0) |
| Ownership |  |
| Public | Ref |
| Private | -11.3 (-16.3; -6.3) |
| Confessional | 6.7 (-1.1.; 14.0) |
| Any type of external support |  |
| No | Ref |
| Yes | 5.8 (1.9; 9.73) |
| Availability of cholera treatment centre |  |
| No | Ref |
| Yes | 13.6 (7.0; 20.2) |
| **Adjusted for facility type, setting, ownership, any type of external support in the previous three years, availability of cholera treatment centre, and province.* | |

**WASH score**

**Supplement Table 18.** Logistic regression analysis of WASH audit score.

| Variable | Adjusted OR*  (95% CI) |
| --- | --- |
| Facility type |  |
| Hospital | Ref |
| Health centre | 0.5 (-8.6; 2.4) |
| Health post | 0.4 (0.2; 1.1) |
| Setting |  |
| Urban | Ref |
| Rural | 0.7 (0.2; 1.7) |
| Ownership |  |
| Public | Ref |
| Private | 0.6 (0.2; 1.5) |
| Confessional | 1.2 (0.3; 5.6) |
| Any type of external support |  |
| No | Ref |
| Yes | 1.8 (0.9; 3.6) |
| Availability of cholera treatment centre |  |
| No | Ref |
| Yes | 1.5 (0.4; 5.3) |
| **Adjusted for facility type, setting, ownership, any type of external support in the previous three years, availability of cholera treatment centre, and province.* | |

**IPC score**

**Supplement Table 19.** Logistic regression analysis of wash IPC score.

| Variable | Adjusted OR*  (95% CI) |
| --- | --- |
| Facility type |  |
| Hospital | Ref |
| Health centre | 0.8 (0.2; 2.9) |
| Health post | 0.1 (0.0; 1.3) |
| Setting |  |
| Urban | Ref |
| Rural | 0.1 (0.1; 0.4) |
| Ownership |  |
| Public | Ref |
| Private | 0.2 (0.1; 0.8) |
| Confessional | 1.0 (0.2; 5.8) |
| Any type of external support |  |
| No | Ref |
| Yes | 1.6 (0.6; 4.5) |
| Availability of cholera treatment centre |  |
| No | Ref |
| Yes | 25.6 (3.5;186.7) |
| **Adjusted for facility type, setting, ownership, any type of external support in the previous three years, availability of cholera treatment centre, and province.* | |
